# Machine Learning in HIV Care and Antiretroviral Therapy: Systematic Review

**DOI:** 10.2196/79219

**Published:** 2026-04-28

**Authors:** Thamina Boudra, Arafate Idrissou, Oussama Barakat, Siamak Davani, Marie-Blanche Valnet Rabier, Jennifer Lagoutte-Renosi

**Affiliations:** 1 Université Marie et Louis Pasteur, CHU Besançon, SINERGIES (UR 4662), Centre Régional de Pharmacovigilance, Besançon, France; 2 Université Marie et Louis Pasteur, SINERGIES (UR 4662), Besançon, France; 3 Université Marie et Louis Pasteur, CHU Besançon, SINERGIES (UR 4662), 19 rue Ambroise Paré, Besançon, F-25000, France

**Keywords:** machine learning, artificial intelligence, HIV, antiretroviral therapy, precision medicine, clinical decision support tools

## Abstract

**Background:**

Artificial intelligence (AI) is expanding across various medical fields, with machine learning (ML) being increasingly used to enhance patient management in diagnosis, prevention, and therapeutic care.

**Objective:**

This study aims to provide an overview of ML applications in HIV care, focusing on real clinical data to improve health care for people living with HIV and on antiretroviral therapy, while highlighting unexplored areas.

**Methods:**

Following PRISMA (Preferred Reporting Items for Systematic Reviews and Meta-Analyses) 2020 reporting guidelines, we analyzed four databases: PubMed, Embase, IEEE, and Web of Science until August 31, 2024. The keywords used were: “Machine Learning,” “HIV,” and “Antiretroviral Therapy.” We excluded from this review studies (1) that were not directly focused on HIV or those that did not apply ML to real clinical data, (2) that focused on pre-exposure prophylaxis, (3) studies involving in silico antiretroviral drug development, and (4) studies on the biological mechanisms underlying HIV diagnosis. Three experts (TB, MBVR, and JLR) screened each article independently.

**Results:**

Overall, 476 studies were identified, and after eligibility assessment, 98 were finally analyzed in detail. Three experts (TB, MBVR, and JLR) identified 6 major categories of ML applications used in the clinical field of HIV: consideration of comorbidities for people living with HIV, predicting drug resistance of the virus, monitoring HIV infection itself, predicting treatment outcomes for people living with HIV, treatment adherence for people living with HIV, and treatment recommendation for clinicians. Random forests emerged as the most used algorithm with 17.49% (43/247), proving effective in identifying biomarkers of metabolic syndrome, genetic features of the HIV envelope, and predicting neurocognitive impairment. Random forests model has several advantages: (1) handle linear, nonlinear data, and missing data, (2) reduce overfitting compared to single trees, (3) robust to noise and outliers, (4) provide feature importance measures, and (5) good generalization ability. Support vector machines demonstrated strong abilities in analyzing the associations between HIV-1 genotypes and resistance phenotypes, predicting virological response to therapy based on HIV genotype, detecting mutations associated with HIV drug resistance , and enhancing computational predictions of resistance from genotype data. Logistic regression appears to be most powerful in predicting various treatment outcomes, including virological failure, adverse events, immune changes in people living with HIV receiving antiretrovirals, and biomarkers of mitochondrial toxicity.

**Conclusions:**

Depending on the field of application, some ML methods are more suitable and adapt better to certain HIV concerns. However, some areas, such as treatment recommendations, treatment adherence, and treatment optimization, still lack AI algorithms and need further exploration, such as therapeutical optimization. The development of new clinical decision-support systems for people living with HIV is the new challenge for the years ahead, and AI represents one of the most promising tools to address it.

## Introduction

Artificial intelligence (AI) has recently gained significant attention due to its growing applications across various domains and the substantial improvements it has brought. Therefore, AI appears to be essential for improving health care [1,2]. A key discipline within AI is machine learning (ML). While AI is defined as the ability of a computer to perform tasks requiring human-like intelligence, ML focuses on developing statistical methods and algorithms that recognize patterns in datasets, improving prediction and classification accuracy when applied to new data. ML's increasing use in health care is justified mainly by the vast amounts of data generated and the ongoing digitalization of the industry, hospitals, and private practices, particularly through the adoption and exploration of electronic health records (EHRs) [3-6]. However, EHRs are not being fully exploited in the European Union due to technical and regulatory obstacles [7]. In response, many research projects were established to enhance the accessibility of EHR data for citizens and researchers in the European Union and facilitate better use [8,9].

Since the emergence of HIV in the 1980s, this disease has remained a major public health challenge, demanding considerable attention from both the scientific community and health care professionals. With advances in antiretroviral treatment, life expectancy for people living with HIV has increased considerably since the 1980s [10].

The management of people living with HIV remains a complex approach and still generates many inquiries from them and health care professionals. Although new therapeutic families have recently been developed, along with new forms such as intramuscular and subcutaneous injections, transforming the management of HIV infection, this only applies to certain eligible people living with HIV. Challenges remain. In addition, people living with HIV now have a near-normal life expectancy, comparable to that of the general population. The management of HIV infection, therefore, continues to pose challenges, particularly in older patients, who often experience age-related comorbidities leading to complex polypharmacy and increased risk of drug-drug interactions (DDI) and adverse drug reactions [11]. Thus, several therapeutic lines could be prescribed across the medical history of people living with HIV. Moreover, with the increasing volume and diversity of data routinely collected as part of people living with HIV care, managing the HIV landscape has become a complex task. This complexity is exacerbated by the heterogeneity of the information, which ranges from clinical data on types of treatment to biological data related to immune status and viral load to viral genetic signatures and socio-demographic factors, all of which require closer examination to improve care for this population.

Thus, comprehensive HIV care could improve both quality of life and long-term health outcomes. In recent years, ML has demonstrated its use in optimizing and revolutionizing various aspects of health care and research. For example, 2 pivotal applications of AI for clinical purposes are rule-based expert systems and clinical decision support systems. Clinical decision support systems combine clinicians’ medical expertise with recent AI advancements to enhance clinical decision-making processes. These systems leverage extensive medical knowledge derived from medical literature, complex algorithms, and patients’ EHR data to assist health care professionals in the overall quality of care while ensuring patient safety. These types of applications are time-consuming and rely on expert consensus. At first glance, it appears that there is a lack of use of ML tools in the field of HIV care. This review aims to identify the main applications of ML in HIV data management and to understand current trends. Specialized vocabulary for nonexperts in AI is summarized in Multimedia Appendix 1.

## Methods

### Search Strategy

An extensive search was conducted across 4 major databases, following the PRISMA (Preferred Reporting Items for Systematic Reviews and Meta-Analyses) 2020 reporting guidelines [12], until August 31, 2024, using a combination of keywords based on a controlled vocabulary thesaurus for indexing articles in each database: “Machine Learning,” “HIV” and “Antiretroviral Therapy.”

### Eligibility Criteria

Studies were included in the current review if they met all the following criteria: titles and abstracts that mentioned explicitly searching terms and using real clinical data. Noninclusion criteria were studies on in silico drug design of antiretroviral therapy (ART), studies on pre-exposure prophylaxis, research focused on the pathophysiology of HIV infection, and studies discussing advancements in HIV clinical diagnostics (Table 1).

**Table 1.** Inclusion and noninclusion criteria.

Category	Inclusion criteria	Noninclusion criteria
Article type	Title and abstract containing search terms: “Machine Learning” and “HIV” and “Antiretroviral Therapy”	Studies on pre-exposure prophylaxis Research focused on the pathophysiology of HIV infection Studies discussing advancements in HIV clinical diagnostics
Language	English	Other languages than English
Population	People living with HIV	—^a^
Study design	Real-world data	In silico drug design of ART^b^

a Not available.

b ART: antiretroviral therapy.

### Study Selection

During the initial screening, 331 records were excluded due to not meeting the research criteria, such as a lack of relevance to HIV or the absence of ML applied to clinical data. Out of 145 articles that advanced to the eligibility phase, 16 were excluded because they were literature reviews or meta-analyses. After successfully retrieving 129 articles, 31 duplicates were eliminated, resulting in 98 articles [6,13-109] that fully met the guidelines and inclusion criteria for this review.

In the clinical field of care for people living with HIV, there are several areas: disease progression, response to treatment, treatment failure, comorbidities, and treatment management. Based on these elements and their reading of the various articles identified, 3 experts (TB, MBVR, and JLR) jointly proposed grouping them into 6 key categories, which are as follows: monitoring HIV infection, predicting treatment outcomes, predicting drug resistance, comorbidities, treatment adherence, and treatment recommendation. Three experts (TB, MBVR, and JLR) screened each article independently. In the event of disagreement, a joint proofreading was carried out to reach a consensus. To facilitate data analysis, we compiled a database that includes the essential details of each study: study title, year of publication, authors, geographical location, and key thematic category, such as comorbidities, prediction of resistance, or treatment adherence, with subcategories for further classification. In addition, we have extracted data on the types of information analyzed (eg, clinical and biological) and the data format (numerical, categorical, and textual). The database also documents study objectives, main results, used AI algorithms and methods, sample size, and authors’ countries of origin. For data trends analysis, concerning the origin of the articles, the origin of the first author was chosen.

### Data Synthesis and Analysis

The data from the included studies were processed and harmonized using customized pipelines. This step involved grouping categories within categorical variables, standardizing formats, and creating additional variables to facilitate the analysis. After data cleaning and preparation, descriptive analyses were conducted to identify key trends and distributions, which were visualized using graphs and figures to provide a clear overview of the collected data. All data processing and analysis were performed in Python using libraries such as pandas, numpy, and matplotlib.

## Results

**Figure 1.**
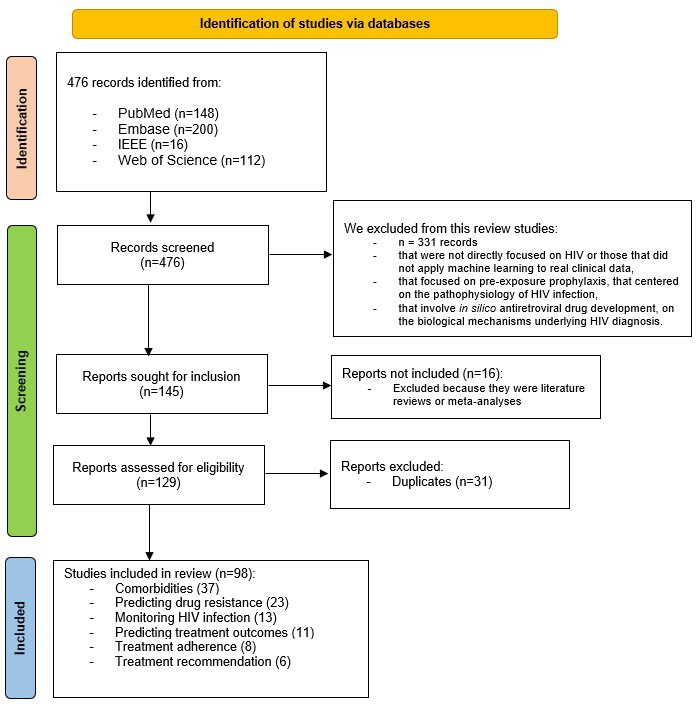
Flow chart process for study selection using the PRISMA (Preferred Reporting Items for Systematic Reviews and Meta-Analyses) 2020 reporting guidelines. N is the number of occurrences.

Overall, 476 studies were collected: 148 from PubMed, 200 from Embase, 16 from IEEE, and 112 from Web of Science (Figure 1).

**Figure 2.**
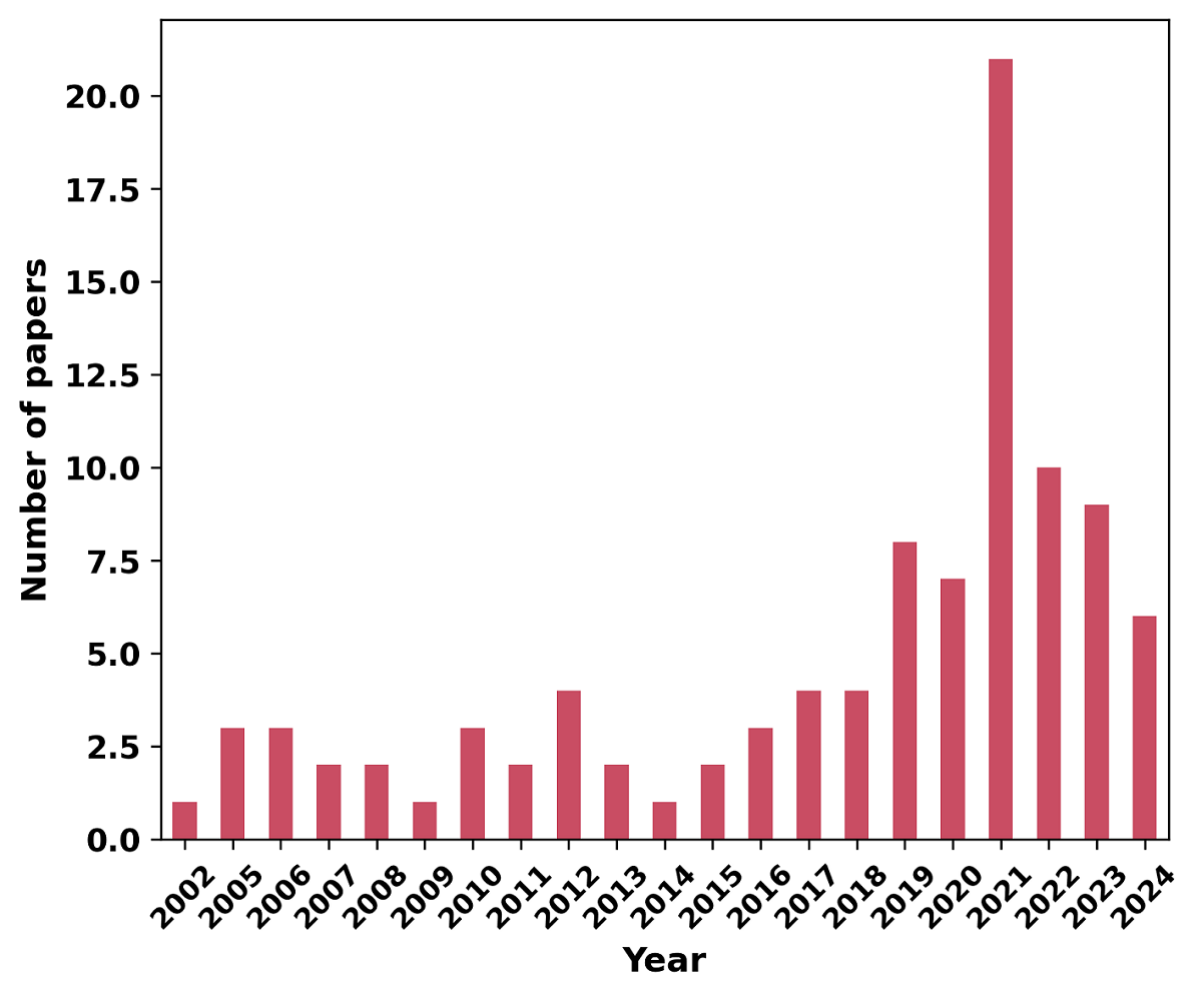
Distributions per year of publications relating to HIV and uses of machine learning over the past twenty years.

We started our analysis with the literature trends by doing a statistical analysis of publication years, which revealed that, on average, articles were published in 2018, with a mean of 2017.7 (SD 5.63) years. The quartiles provide additional insight: the first quartile indicates that 25% (24/98) of articles were published before 2015. The median year of publication is 2020 (IQR 6.75-7), meaning that half the articles were published after this date. Finally, 25% (25/98) of articles were published after 2020, highlighting a clear trend in favor of more recent studies. These results reflect a strong temporal coverage of our literature review. Regarding the distribution of papers over time, the earliest study on HIV treatment using AI was published in 2002. Although publications remained sporadic until 2005, a noticeable uptick occurred afterwards (Figure 2).

**Figure 3.**
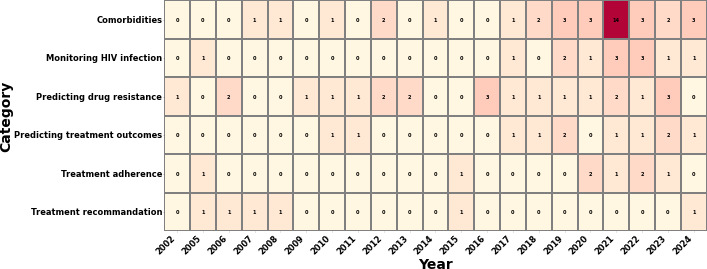
Annual distribution of publications by category.

Figure 3 illustrates the number of articles published in each category since 2002. For the comorbidities category, the first article was published in 2007. Notably, there were no publications in 2009, 2011, 2015, and 2016, with a peak of 14 articles published in 2021, indicating growing interest and progress in this area. In the HIV infection monitoring category, the first article appeared in 2005, followed by a second one after a 10-year gap. In the category of predicting drug resistance, the first article was published in 2002, marking the earliest of the selected articles. It took 4 years for the second publication, with 3 articles appearing in 2016 and 2023. The first article on predicting treatment outcomes was published in 2010. In the treatment adherence category, the first article was in 2005, with a 20-year gap before the second publication. Finally, in the treatment recommendation category, 4 articles were published between 2005 and 2008. This trend saw a significant surge post-2015, culminating in a peak in 2021. The 2021 peak may have been influenced by the COVID-19 pandemic, which intensified global research efforts and resulted in a higher volume of publications (Table S6 in Multimedia Appendix 2).

Among the 6 broad categories described above, the number of publications devoted to comorbidities amounts to 38% (n=37), closely followed by studies on the prediction of drug resistance at 23% (n=23), then monitoring HIV infection itself at 13% (n=13), predicting treatment outcomes at 11% (n=11), treatment adherence at 8% (n=8), and treatment recommendation at 6% (n=6).

**Figure 4.**
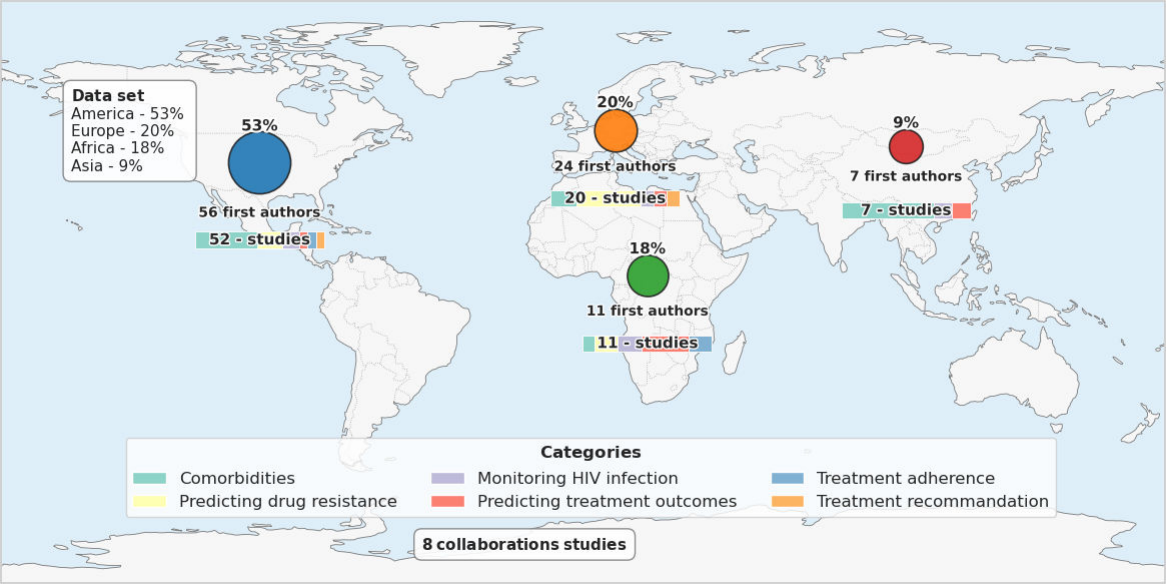
Sources of datasets and number of studies per continent, with number of first authors across regions. For each continent, a mini bar chart details the distribution of studies into six categories (comorbidities, prediction of drug resistance, HIV infection monitoring, prediction of treatment outcomes, treatment adherence, and treatment recommendations).

**Figure 5.**
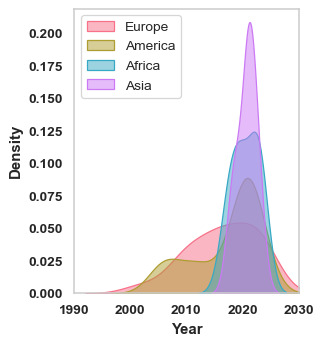
Density estimation of articles per year. Articles are assigned to a continent based on the affiliation of the first author. Europe (red), America (brown), Africa (blue), and Asia (purple).

More than half of the data used in the included studies came from America (more than 50% [53/98]), followed by Europe (20/98, 20%), Africa (18/98, 18%), and Asia (9/98, 9%). This imbalance revealed a strong dominance of data from Western regions, while data from Africa and Asia remained underrepresented. Overall, 52 studies were conducted in America [6,13-63], 20 in Europe [64-83], 11 in Africa [84-94], and 7 in Asia [95-101]. In addition, 8 studies are classified as collaborative [102-109], as the authors’ affiliations span multiple continents. The number of first authors is 56 in America [6,13-63,102,103,107,108], 24 in Europe [64-83,104-106,109], 11 in Africa [84-94], and 7 in Asia [95-101] (Figure 4). Interestingly, the density of articles by region showed a slightly different distribution (Figure 5).

**Figure 6.**
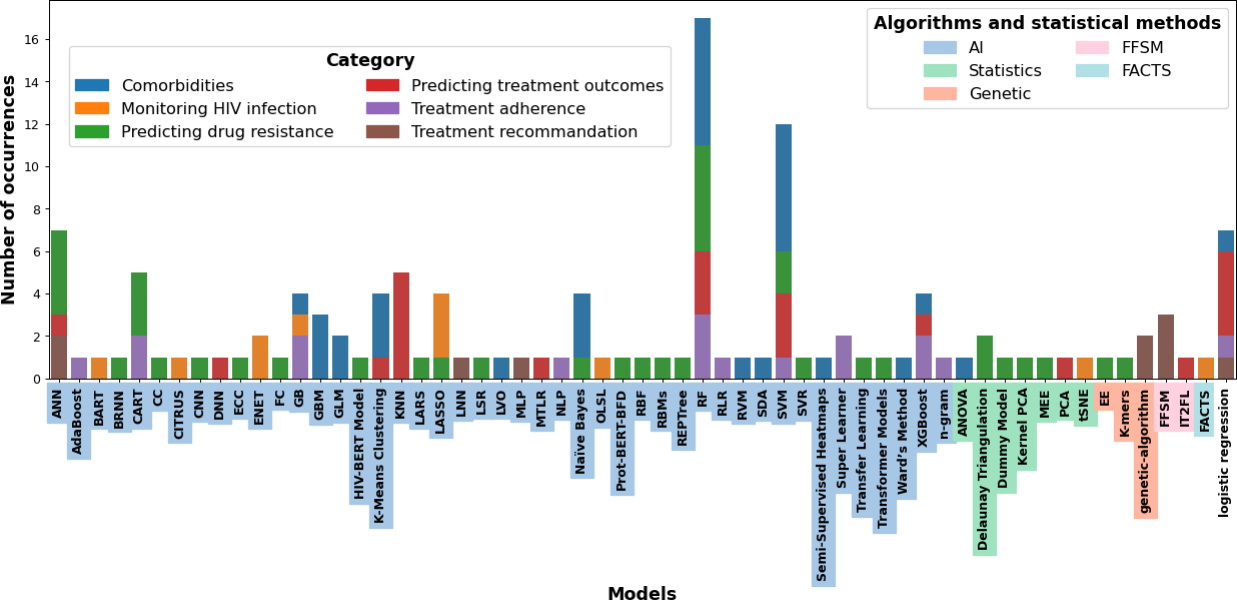
Most used algorithms and statistical methods in publications relating to HIV and uses of machine learning. This figure presents the methods used in the different included articles, along with their frequency of occurrences across each of the six categories. Additionally, it highlights their respective affiliations. AI: artificial intelligence; FFSM: fuzzy finite state machine models; FACTS: fairness-aware causal paths decomposition.

Fairness-aware causal paths decomposition around 2020, Africa and Asia presented the highest concentration of research activity on this topic, with Asia reaching the most significant peak, followed by Africa. Europe and America also make notable contributions, but with less intensity. The datasets used in these studies come from a variety of countries and are highly heterogeneous. To deepen the analysis, the various data types used in the reviewed articles are summarized in Figure 6.

**Figure 7.**
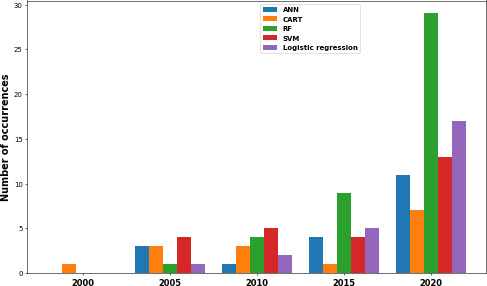
Algorithms and statistical method temporal trend (between 2000 and 2020) relating to HIV and uses of machine learning. Artificial neural network (blue), classification and regression trees (orange), random forest (green), support vector machine (red), and logistic regression (purple). ANN: artificial neural network; CART: classification and regression trees; RF: random forest; SVM: support vector machine.

The three main data types are first numerical (n=75), then categorical (n=69), and finally textual (n=3). The 5 most used databases are cohort data (n=70), the Stanford database (n=15), the EuResist database (n=4), the Los Alamos National Laboratory database (n=3), and the Akwa Ibom database (n=2) (from a southern Nigerian state). Of these, HIV cohort data are the most widely used, particularly in studies of comorbidities, predicting drug resistance, predicting treatment outcomes, monitoring HIV infection, treatment adherence, and treatment recommendations. The Stanford database, which specializes in HIV drug resistance, comes second and is mainly used for research into resistance mutations. Finally, the EuResist database, the fruit of collaboration between several European sources, is used in 4 separate studies. A wide range of ML statistical methods were used in the studies we reviewed. The most used methods are random forest (RF), support vector machine (SVM), and logistic regression, each playing a key role in different aspects of HIV care research (Figure 7). Regarding method and algorithm trends, RF emerges as the most widely used algorithm, with 17.49% (43/247), reflecting its growing popularity in recent years. RF appears around 2010, with increasing use and peaking significantly in 2021. Artificial neural network (ANN), SVM, and logistic regression appeared after 2005, with increased use since 2017.

**Figure 8.**
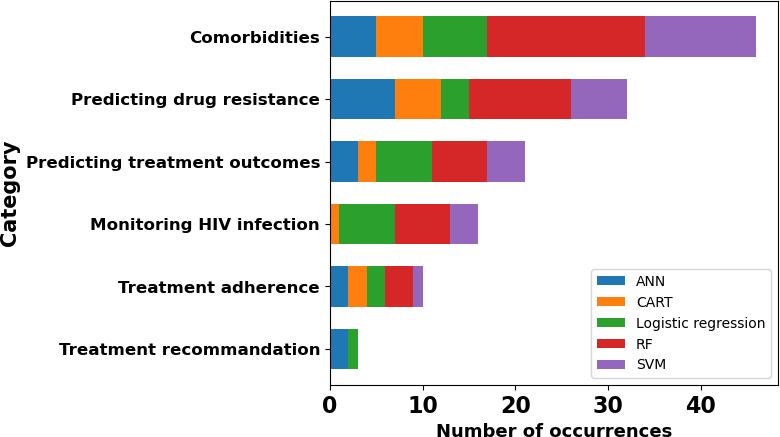
Five most used algorithms and statistical methods relating to HIV and uses of machine learning for each category. Artificial neural network (blue), classification and regression trees (orange), random forest (green), support vector machine (red), and logistic regression (purple). ANN: artificial neural network; CART: classification and regression trees; RF: random forest; SVM: support vector machine.

An in-depth analysis of the most used ML methods in the 6 categories mentioned above (Figure 8), as well as the tasks for which they are most effective, is detailed in this section. Logistic regression appears in all categories, whereas the ANN model was not used in monitoring HIV infection, and the other 3 methods were not used in the treatment recommendation category. We can observe that RF is the most used algorithm in each category where it appears. SVM is the second most widely used algorithm within the comorbidities category, ANN is used in predicting drug resistance, and logistic regression is used in predicting treatment outcomes and monitoring HIV infection.

In the comorbidity category [6,13-35,61,64-67,84,95-99,102,103] (Figure S1 in Multimedia Appendix 3), which constitutes the largest part of our study data (37/98, 38%; Figure 4), ML methods were used for various tasks, including identifying risk factors for cardiac complications, predicting vaccine responses in the context of HIV co-infection, testing for retinal damage in people living with HIV that may lead to subtle visual field defects, and predicting increasing comorbidity risks using EHR data [6]. Cardinal [13] conducted a study that identified significant associations between carotid artery plaques and selected features in a population of HIV-positive and HIV-negative individuals with low to intermediate cardiovascular risk. The RF algorithm effectively distinguished between individuals with and without carotid artery plaques by combining traditional cardiovascular risk factors with strain elastography features, using 5 most discriminant combinations of features achieved area under the receiver operating characteristic (AUROC) between 0.76 and 0.80 as classification performance. For predicting vaccine response in the context of coinfection, nonlinear models estimated by regression tree and RF were more accurate than generalized linear models in predicting humoral responses to SARS-CoV-2 mRNA vaccination in people living with HIV (classification and regression trees: R^2^=0.795; root mean square error=0.451 and RF: R^2^=0.845; root mean square error=0.412) [64]. This is linked to the fact that the different feature selection strategies used in linear models often tend to exclude important variables which, taken in isolation, might have a more significant predictive role [110]. Kozak et al [14] and Goldbaum et al [15,61] investigated whether people living with HIV had retinal lesions resulting in subtle visual field defects, and whether ML classification methods could distinguish these defects from those of HIV-negative individuals. The results confirm that HIV has an effect on the retina, and the eyes of people living with HIV with low cluster of differentiation 4 (CD4) counts show visual field defects and retinal lesions, while those of people living with HIV with high CD4 counts can appear normal. The SVM method was able to distinguish the visual fields of people living with HIV from those of HIV-negative people, even for people living with HIV with high CD4 counts, a task that is more challenging for a human expert. The model achieved an AUROC of 0.843 for the subset of people living with HIV with low CD4 counts, and an AUROC of 0.695 for those with high CD4 counts. Another study was also conducted on predicting the risk of multidrug-resistant enterobacterial (MDR-E) infections among people living with HIV [16]. Among 4734 study participants, MDR-E was isolated from 1.6% (95% CI 1.2%‐2.1%). In unadjusted analyses, MDR-E was strongly associated with nadir CD4 cell count ≤200 cells/mm^3^ (prevalence ratios [PR], 4.0; 95% CI, 2.3‐7.4), history of an AIDS-defining clinical condition (PR, 3.7; 95% CI 2.3‐6.2), and hospital admission in the prior 12 months (PR, 5; 95% CI 3.2‐7.9). Searches were also made to identify biomarkers and assess the patterns of T lymphocyte cell activation associated with the development of tuberculosis following the initiation of ART in people living with HIV displaying high CD4 counts [95,102]. Furthermore, ML methods were able to identify biomarkers associated with metabolic syndrome and HIV-associated neurocognitive disorders. They also pinpointed genetic features on the HIV envelope, accurately predicted neurocognitive impairment, and recognized molecular signatures linked to HIV-associated neurocognitive disorders diagnosis [17-23,25,29-31,33-35,65,84,96,97,103].

Another example of application: a multivariable logistic regression model was more suitable to predict the risk of serious falls among an older population of people living with HIV. However, including ART classes taken by these individuals did not improve the algorithm’s prediction, with the C-statistic increasing only slightly from 0.725 to 0.732 after the inclusion of ART classes [32]. The most frequently used method in this category is RF, as seen in (Figure S1 in Multimedia Appendix 3), valued for its robustness and use of the bootstrap principle.

For predicting or classifying drug resistance to antiretrovirals and identifying genetic signatures, 23% (23/98) of the articles focused on this area (Figure 4; Table S2 in Multimedia Appendix 2; Figure S2 in Multimedia Appendix 3) [36-43,62,63,68-77,85,86,104]. The classification and regression trees algorithm was able to predict drug resistance phenotypes from HIV-1 genotypes with good accuracy; some key sequence positions that are associated with drug resistance were identified using a mutual information analysis [68]. RF algorithm shows strongest correlation between predicted and actual virological responses, outperforming SVM and ANN. The mean absolute error ranged in [0.494-0.644] for RF, in [0.50-0.790] for SVM, and in [0.677-0.903] for ANN. Combining these methods further enhanced prediction accuracy, indicating that ensemble approaches are particularly effective in forecasting virological response [62]. ML methods were also used to analyze correlations between HIV-1 genotypes and resistance phenotypes, to predict virological responses to therapy based on HIV genotype, to detect mutations associated with HIV drug resistance without requiring expert knowledge, and to improve computational prediction of resistance from genotype data [36-43,63,69-77,85,86,104]. Both RF and SVM classified reverse transcriptase mutants with known resistance or sensitivity to nevirapine, achieving high accuracy in predicting susceptibility to the drug.

In the category of monitoring HIV infection, 13% (13/98) of (Table S3 in Multimedia Appendix 2; Figure S3 in Multimedia Appendix 3) studies involved applications of ML for tracking viral load and infection status [44-50,78,79,87,88,100,105]. For example, some studies identify biomarkers of viral rebound in people living with HIV from independent cohorts' pretreatment interruption [47] or tend to determine the viral compartmentalization dynamics to document a specific genetic signature of virus [44]. Mahto and Sood [49] evaluate various ML methods for predicting HIV progression and patient outcomes, identifying RF and extreme gradient boosting as the most effective, with accuracy rates of 0.88 and 0.89, respectively. They highlight the potential for further optimization through hyperparameter fine-tuning, feature engineering, and incorporating additional data sources to enhance algorithm robustness and generalizability. Kagendi and Mwau [88] developed an RF algorithm to predict HIV viral load hotspots in Kenya as an early warning system, achieving an accuracy of 0.78 and correctly identifying 434 viral load hotspots in December 2019, demonstrating its potential to optimize ART programs by supporting proactive resource allocation. A study found that the risk of HIV infection was higher for African Americans compared to non-African Americans. By using the fairness-aware causal paths decomposition method, researchers identified several social determinants of health that contribute to this racial disparity, including education, income, violent crime, drinking, smoking, and rurality. Performance evaluation of predictive models demonstrated comparable results across methods: boosted logistic regression achieved an AUROC of 0.79, decision tree-based methods yielded an AUROC of 0.77, and RF outperformed with an AUROC of 0.80 [50]. The SVM was the top-performing algorithm for predicting CD4/CD8 ratios in patients with baseline CD4 counts below 200 cells/ml. The RF followed with R^2^ values of 0.438 and 0.519 for SVM [100]. ML methods also monitored ART adherence and retention in care for people living with HIV.

For predicting treatment outcomes, 11% (11/98) of the articles explored this topic (Table S4 in Multimedia Appendix 2; Figure S4 in Multimedia Appendix 3) [51-53,80,81,89-92,101,106]. Among 7 classifiers, the RF algorithm achieved high accuracy in predicting CD4 count changes, with precision, sensitivity, and recall values close to 0.99 (sensitivity=1.00, precision=0.987, *F*_1_-score=0.993, AUC=0.998), and it outperformed other algorithms in predicting virological failure and identifying key predictors [89]. The logistic regression excelled in predicting virological suppression [90]. ML methods were also able to predict adverse events [53], immune changes in ART recipients [52,54], biomarkers linked to mitochondrial toxicity [51,52], viral load [81,92], and highly active antiretroviral therapy (HAART) regimens recommendations [80].

For treatment adherence and predicting retention in care, 8% (8/98) of the articles examined ML applications (Table S5 in Multimedia Appendix 2; Figure S5 in Multimedia Appendix 3) [54-57,93,94,107,108]. A study conducted in Nigeria by Ogbechie et al [94] was able to predict interruptions in ART treatment at 30 days among people living with HIV, using routine program data. The method used integrated boosting tree and extreme gradient boosting techniques and demonstrated a strong performance, with a sensitivity of 0.81, specificity of 0.88, and a positive predictive value of 0.83. After its integration into the national electronic medical records system, the proportion of people living with HIV interrupted ART treatment cases decreased from 58.6% to 14.2%. Health workers reported that the model enabled proactive interventions, underscoring the potential of ML to enhance HIV treatment adherence and improve patient outcomes. Stockman et al [108] used ML methods to predict loss to follow-up among people living with HIV on ART treatment in Nigeria and Mozambique, using data from health facilities, geospatial sources, and satellite imagery. For the Mozambique dataset, the RF was the best method with an area under the precision-recall curve of 0.65, while in Nigeria, the boosted tree method achieved an area under the precision-recall curve of 0.52. Lu et al [57] demonstrated that SVM effectively models factors influencing adherence to HAART in people living with HIV, outperforming neural networks.

In total, 6 articles (6%) [58-60,82,83,109] discussed the use of ML for treatment recommendations in people living with HIV (Table S6 in Multimedia Appendix 2; Figure S6 in Multimedia Appendix 3. Fuzzy discrete event system-based HIV regimen selection system demonstrated high self-learning accuracy in predicting treatments, with more than 80% agreement with actual prescribed regimens for 35 patients under practical conditions. The system outperformed neural networks in transparency and interpretability and showed an accuracy of 0.84 to 1 in predicting new treatment regimens. Additionally, it achieved 82.9% agreement with non-expert physicians and 100% agreement with expert physicians. By considering clinical, demographic, and genotypic data, the combined system provided the best prediction performance for HAART therapy recommendations based on genotype (Figure S6 in Multimedia Appendix 3) [58-60,82,109]. Another approach was to assess the relevance of clinical drug interaction (DDI) with ART. Two DDI prediction algorithms: DeepARV-ChemBERT and DeepARV-sim were developed by Pham et al [83] for predicting DDIs between ARVs and comedications. Those algorithms achieved a weighted accuracy of 0.729 and 0.776, respectively.

## Discussion

### Principal Findings

Through our scoping review, we identified 6 major categories of ML applications used in the clinical field of HIV: consideration of comorbidities for people living with HIV, predicting drug resistance of the virus, monitoring HIV infection itself, predicting treatment outcomes for people living with HIV, treatment adherence for people living with HIV, and treatment recommendation for clinicians. In terms of models, RF emerged as the most used algorithm in all of the studies cited in this review.

Our results indicate that certain ML models are better suited to certain tasks in the field of HIV care. Indeed, model performance depends on the type and complexity of the data. In studies predicting the drug resistance of the virus, the data comes from the 2 main sources, namely the Stanford HIV Drug Resistance Database and EuResist DataBase. Thus, we observed that some models work better with linear relationships, while others, such as RFs, can handle both linear and nonlinear data, which may explain their widespread use in many of the articles included in this review.

### Limitations

This scoping review presents a few limitations, relative to the review process itself. First, non-English articles have been excluded, which may omit some ML applications, but English remains the major language of scientific publications, and therefore, this bias appears to be limited. Second, this literature analysis did not assess in a comprehensive manner the methodological quality of the included studies, which represents a possible bias in the interpretation. To address these limitations, next research must consider in detail the robustness of the different approaches used in those studies in order to propose best practices and specific limitations of AI models used in the field of HIV. However, our main goal was to map the existing publications using AI in the field of HIV. In Table 2, we discuss the strengths and limitations of the various ML tools identified in this literature analysis on people living with HIV care.

**Table 2.** Strengths and limitations of the most widely used machine learning algorithms.

Algorithms	Applications	Strengths	Limitations
Random forest (RF)	Effectively distinguished between individuals with and without carotid artery plaques Predicting vaccine response in the context of coinfection Predicting (forecasting) virological response Classified reverse transcriptase mutants with known resistance or sensitivity to nevirapine Predict HIV progression and patient outcomes Predict HIV viral load hotspots Identified several social determinants of health contributing to this racial disparity Predict CD4^a^ count changes Predict humoral responses	Can handle linear, nonlinear data, and missing data Reduce overfitting compared to single trees Robust to noise and outliers Provide feature importance measures Good generalization ability	Less interpretable than a single tree Can be computationally heavy with large datasets Struggles with very high-dimensional sparse data
Support vector machines (SVM)	Able to distinguish the visual fields of people living with HIV from those of HIV-negative people Predict risk of MDR-E^b^ infections among people living with HIV Classified reverse transcriptase mutants with known resistance or sensitivity to Nevirapine Predict (forecasting) virological response Effectively models factors influencing adherence to HAART^c^	Effective in high-dimensional spaces Works well with clear margin of separation. Kernel trick allows nonlinear classification	Not scalable to very large datasets. Requires careful kernel and parameter choice Less interpretable than simple models
Decision trees (CART)^d^	Predict drug resistance phenotypes from HIV-1 genotypes with good accuracy Identified several social determinants of health contributing to this racial disparity Effectively models factors influencing adherence to HAART and identifying genetic signatures	Easy to visualize and interpret Handles nonlinear relationships and interactions naturally Can work with categorical and numerical data No need for feature scaling	Unstable with small data changes Prone to overfitting Not as accurate as ensemble methods
Artificial neural networks (ANN)	Predict drug resistance phenotypes from HIV-1 genotypes with good accuracy Identified several social determinants of health contributing to this racial disparity Effectively models factors influencing adherence to HAART and identifying genetic signatures	Capture highly complex and nonlinear patterns Can automatically learn feature representations	Require massive amounts of data High computational Hard to interpret (black box) Risk of overfitting without regularization Training can be unstable
Logistic regression	Predict drug-drug interaction Predict drug resistance Predict virological suppression Predict the risk of serious falls among an older population of people living with HIV	Simple, fast, and interpretable Works well with small datasets Easy to implement and regularize	Assumes linear relationships between predictors and outcome Struggles with complex and nonlinear data Sensitive to multicollinearity and outliers Limited predictive power compared to advanced models

a CD4: cluster of differentiation 4.

b MDR-E: multidrug-resistant enterobacterial.

c HAART: highly active antiretroviral therapy.

d CART: classification and regression trees.

We can also consider certain limitations inherent in the results. Concerning the type of AI models, a lot of articles focus on ML models, while other AI approaches, such as natural language processing and large language models have been less explored or remain underexplored. Moreover, data availability is limited: many studies rely on small or localized datasets, with frequent reuse of the same databases, reducing both novelty and representativeness. Databases dealing with the issue of AI applied to HIV are heterogeneous and require the definition of a federated database oriented by clinical field. This highlights the need to develop large-scale, interconnected, multicenter, and openly accessible datasets in order to reduce barriers to progress, for example, to improve the performance of causal inference methods.

We could similarly address as limitations the complexity of this disease. For example, another chronic disease such as type 2 diabetes mellitus [111] revealed common difficulties, in particular in the lack of model validation across different clinical contexts and populations. Indeed, López et al [111] also expose the necessity to build highly specialized and effective AI models. In a similar way, algorithms trained on specific subpopulations of people living with HIV may not provide the same performance score with other different populations of people living with HIV due to genetic variations, lifestyles, and socioeconomic factors. That is why, as in clinical trials, the characteristics of individuals are crucial, as they determine whether or not the results can be extrapolated.

The use of AI in health care raises important ethical questions, particularly with regard to patient data privacy and informed consent. Regulations governing personal data are not standardized, and the data currently available is not structured in the same way and is often incomplete, which can limit its use with AI methods and tools. The establishment of clear, globally standardized regulatory frameworks and training in data ethics for health care professionals and students is essential to ensure the safe, ethical adoption, trust of AI, and the development of sustainable solutions for the care of people living with HIV.

### Future Directions

We observed through this scoping review that many applications of ML are not yet explored. Among these is the field of public health, where identifying clusters through social media monitoring would enable contact tracing. AI methods and tools could also be used for individual monitoring, disease progression, and real-time support for people living with HIV. Notably, this review did not identify any studies on predicting the onset of adverse effects and therapeutic optimization in the context of drug resistance, DDI, and iatrogenic events, as well as other associated chronic diseases that must incorporate the concept of aging. Among these areas of research, monitoring adverse effects is becoming an important field. The field of AI in pharmacovigilance is gaining momentum in health care [112-114]. Recently, several studies have focused on predicting HIV-related mortality and comorbidities, or predicting admission to intensive care [115-118].

Another point to consider for the future is the use of algorithms based on real clinical data that could be used serving a teacher and assessor in medical education, with the creation of fictitious patients, for example, to improve management by future clinicians. This should help to educate practitioners about AI.

### Conclusions

In summary, our review identified the most suitable ML methods across the 6 categories chosen. RF emerged as the most used algorithm due to its versatility and suitability for both classification and prediction tasks. Following closely was the SVM, both of which are supervised ML algorithms widely applied to various aspects of HIV infection challenges. The diversity of databases is a critical element to consider. However, key challenges remain, including limited data availability, quality, and accessibility, which continue to hinder the broader adoption of evidence-based medicine in HIV care. Regular updates of ML tools are crucial to ensure that evidence-based medicine tools provide accurate, up-to-date information for clinical decision-making. Although there has been an increase in publications across various aspects of HIV care, there remains a critical lack of research addressing treatment safety and optimization. Future efforts should focus on monitoring adverse events and developing decision-support tools based on the risk of drug-related adverse effects. Addressing this gap will be the focus for future studies, where the aim to leverage AI is to develop more personalized, effective ART regimens.

## Supplementary material

10.2196/79219 Multimedia Appendix 1 Artificial intelligence vocabulary - some basic definitions.

10.2196/79219 Multimedia Appendix 2 Selected studies with their summaries for each category.

10.2196/79219 Multimedia Appendix 3 Top five algorithms and statistical methods for all categories.

10.2196/79219 Checklist 1 PRISMA 2020 checklist.

## Abbreviations

AI: artificial intelligence
ANN: artificial neural network
ART: antiretroviral therapy
AUROC: area under the receiver operating characteristic curve
CD4: cluster of differentiation 4
DDI: drug-drug interaction
EHR: electronic health record
HAART: highly active antiretroviral therapy
MDR-E: multidrug-resistant enterobacterial
ML: machine learning
PR: prevalence ratio
PRISMA: Preferred Reporting Items for Systematic Reviews and Meta-Analyses
RF: random forest
SVM: support vector machine
