# Supplementary material for: Machine Learning in HIV Care and Antiretroviral Therapy: Systematic Review
Source: J Med Internet Res. 2026 Apr 28;28:e79219. doi: 10.2196/79219 (PMC13123759; doi:10.2196/79219)
Supplement: Multimedia Appendix 1 [file jmir-v28-e79219-s001.docx]

| **Vector** | An ordered list of numbers that can represent a point in space or a set of features describing an object or observation |
| --- | --- |
| **Feature** | The input variable used by a machine learning method to make predictions or classifications |
| **Validation set approach** | The process of randomly dividing the available set of observations in a training set (to fit the model) and a validation set (to build predictions) to evaluate the predictive model’s performances |
| **Machine Learning** | Teaching computers to recognize patterns in data and make decisions or predictions based on those patterns |
| **Unsupervised ML** | Algorithm that learns patterns from unlabeled data without explicit guidance |
| **Supervised ML** | Algorithms that learn from labeled data to make predictions or classifications |
| **Naïve Bayes Algorithm** | A supervised classification learning algorithm |
| **Gaussian naïve bayes** | A variant of Naïve Bayes dealing with continuous data and assuming that the continuous values associated with each class are distributed according to a Gaussian distribution; it is well-known for its effectiveness and efficiency in multiclass prediction |
| **Logistic Regression** | Machine Learning model for classification problems |
| **Decision trees and CART algorithm** | A non-parametric supervised learning method for regression and classification tasks consisting in stratifying the feature space into regions; prediction for a given observation is obtained by averaging the observations belonging to the same region; CART algorithm is one of the most used splitting criteria |
| **Random forest RF** | An ensemble learning method for classification and regression tasks consisting in creating many randomized decision trees and aggregating their predictions by averaging. |
| **Neural network NN** | Computational systems composed of interconnected nodes (called "neurons") organized in layers. These networks are designed to recognize patterns, process complex data, and make decisions or predictions |
| **ANN; DNN** | ANN (Artificial Neural Network); DNN (Deep Neural Network) |
| **Support Vector machine SVM** | Supervised machine learning algorithm that finds the optimal boundary (hyperplane) to separate different classes of data points |
| **Adaptive boosting (AdaBoost)** | An ensemble classification learning method that iteratively builds a series of weak classifiers, focusing on the misclassified examples from previous iterations, and combines them into a final strong classifier. |
| **K- nearest Neighbor KNN** | Supervised machine learning method for classification and regression tasks that classifies, respectively predicts, the value of an observation based on the majority vote, respectively the average, of its k nearest neighbors in the feature space |
| **Multilabel classification chain MLC** | An algorithm that transforms a multilabel problem into a series of binary classification problems, where each classifier in the chain predicts a single label and uses the predictions of previous classifiers as additional input features |
| **LASSO regression** | Regularization method of the regression coefficient estimates of a linear model to improve prediction accuracy with (multi)-collinearity among the independent variables |
| **Linear discriminant analysis** | Supervised learning method for classification tasks |

**Artificial intelligence vocabulary - some basic definitions**
