# Supplementary material for: Machine Learning in HIV Care and Antiretroviral Therapy: Systematic Review
Source: J Med Internet Res. 2026 Apr 28;28:e79219. doi: 10.2196/79219 (PMC13123759; doi:10.2196/79219)
Supplement: Multimedia Appendix 2 [file jmir-v28-e79219-s002.docx]

**Selected studies with their summaries for each category**

**Table S1.** Comorbidities

| **Year** | **Author** | **Ref.num** | **Title** | **Purpose** | **Findings** |
| --- | --- | --- | --- | --- | --- |
| 2007 | Kozak et al | 14 | Machine learning classifiers detect subtle field defects in eyes of HIV individuals. | To test if the eyes of patients with human immunodeficiency virus (HIV) have retinal damage that causes subtle field defects and to test if sensitive machine learning classifiers are able to distinguish fields in HIV and non-HIV subjects using these field defects | Eyes of HIV patients with low CD4 counts have retinal damage and visual field defects that can be detected using sensitive machine learning classifiers. those defects were mostly located superiorly near the blind spot, suggesting damage to the retina inferiorly near the optic disc. In HIV patients with high CD4 counts, sensitive machine learning classifiers can distinguish their visual fields from those of normal, HIV-negative individuals, despite the fields appearing normal. |
| 2008 | Goldbaum et al | 61 | Analysis with support vector machine shows HIV-positive subjects without infectious retinitis have mfERG deficiencies compared to normal eyes | To test if eyes from individuals with human immunodeficiency virus (HIV) have electrophysiologic abnormalities that manifest as multifocal electroretinogram (mfERG) abnormalities. And if they can help distinguish HIV from normal eyes, using SVM | The mfERG is a sensitive detector of the effect of HIV on the retina, even in the high-CD4 group; MLCs can maximize the class information that can be obtained; HIV-positive patients with no history of immune suppression may be damaged because of the presence of the HIV virus; and there is no observed pattern to the retinal effects from HIV detected by the SOK of mfERG. |
| 2011 | Goldbaum et al | 15 | Pattern recognition can detect subtle field defects in eyes of HIV individuals without retinitis under HAART | To use machine learning classifiers (MLCs) to seek differences in visual fields (VFs) between normal eyes and eyes of HIV+ patients | Eyes from both low and high CD4 HIV+ patients have visual field defects indicating retinal damage.  - Machine learning classifiers like SVM and standard perimetry measures like MD are effective at detecting these subtle visual field defects in HIV-positive patients.  - The SVM and MD classifiers were able to significantly distinguish the visual fields of HIV-positive patients with low CD4 counts from normal controls. |
| 2012 | Holman et Gabuzda | 17 | A Machine Learning Approach for Identifying Amino Acid Signatures in the HIV Env Gene Predictive of Dementia. | Using machine learning approach to identify HIV genetic signatures correlated to HIV associated dementia | The PART algorithm identified 5 signatures associated with HAD diagnosis that were evaluated in 2 independent datasets, 2 signatures were validated in HIV env sampled from the CSF. |
| 2012 | Choi et al | 18 | Genetic features of cerebrospinal fluid-derived subtype B HIV-1 tat. | To identify the viral characteristics of HIV-1 subtype B tat that are associated with viral populations in the blood and CSF and neurocognitive dysfunction. | The nucleotide position (HXB2 position 5905) within the cysteine-rich domain appears to be a signature of the viral populations derived from the CSF HIV population diversity of that seems to be higher in subjects with HAND and a genetic between the blood and CSF derived that was not associated with neurocognitive impairment, it was inversely correlated with current and nadir CD4+ T cell counts. |
| 2014 | Cassol et al | 19 | Cerebrospinal fluid metabolomics reveals altered waste clearance and accelerated aging in HIV patients with neurocognitive impairment. | To profile and identify cerebrospinal fluid biomarkers associated with HAND in PLWH | Machine learning methods identified alteration is CSF metabolome and eight metabolites are designated as top-ranked classifiers of HAND, including neurotransmitters (glutamate, N-acetylaspartate), markers of glial activation (myo-inositol), ketone bodies (betahydroxybutyric acid, 1,2-propanediol) and mitochondrial function (succinate) |
| 2017 | Underwood et al | 65 | Gray and White Matter Abnormalities in Treated Human Immunodeficiency Virus Disease and Their Relationship to Cognitive Function. | To establish the relationship between cognitive impairment and brain structure in successfully treated patients using multimodal neuroimaging | Cognitive impairment, lower gray matter volume, and white matter microstructural abnormalities were evident in in in PLWH under suppressive ART, cognitive impairment was associated with White matter abnormalities. |
| 2018 | Ogishi and Yotsuyanag | 96 | Prediction of HIV-associated neurocognitive disorder (HAND) from three genetic features of envelope gp120 glycoprotein**.** | To predict HAND status using machine learning approaches | Machine Learning Classifier accurately identified three genetic features of HIV env gene (amino acid positions in gp120 glycoprotein) that were predictive of HAND status. combining these three genetic features enabled them to identify four HAND clusters and could help develop new cART optimized for HAND quasispecies. |
| 2018 | Underwood et al | 20 | Multivariate Pattern Analysis of Volumetric Neuroimaging Data and Its Relationship With Cognitive Function in Treated HIV Disease | To predict neurocognitive impairment in PLWH using only volumetric neuroimaging data by building a multivariate model. | To predict neurocognitive impairment in PLWH using only volumetric neuroimaging data by building a multivariate model. |
| 2019 | Babu et al | 97 | Plasma Metabolic Signature and Abnormalities in HIV-Infected Individuals on Long-Term Successful Antiretroviral Therapy | To understand the plasma metabolomic changes and predict the risk of accelerated aging in PLHIV on long-term suppressive antiretroviral therapy (ART) | PLHIV on long-term antiretroviral therapy has significant metabolic abnormalities compared to healthy controls, including lower levels of essential amino acids, altered energy metabolism, and changes in phospholipids and complex lipids. The metabolic changes in PLHIV indicate a higher risk of inflammatory and neurological diseases. The metabolic abnormalities persist even after long-term suppressive antiretroviral therapy, suggesting ongoing chronic inflammation and oxidative stress in PLHIV. |
| 2019 | Luckett et al | 21 | Deep Learning Analysis of Cerebral Blood Flow to Identify Cognitive Impairment and Frailty in Persons Living With HIV. | To use DNN in order to classify PLWH cognitive impairment and frailty in PLWH based on a battery of tests | The DNN models used successfully classified cognitive impairment and frailty status in PLWH. Feature selection algorithms identified the predictive regions in each domain: frailty in HIV is primarily subcortical and cognitive impairment in HIV involves subcortical and cortical regions. |
| 2019 | Dastgheyb et al | 22 | Cognitive Trajectory Phenotypes in Human Immunodeficiency Virus-Infected Patients. | To categorize longitudinal changes in HIV-infected patients based on the performance in specific cognitive domains. | Four distinct cognitive change phenotypes were identified  Decline in verbal fluency  Decline in executive function  Declines in Learning and Recall  Declines in Motor Function |
| 2020 | Jaganath et al | 102 | Evaluation of multi-antigen serological screening for active tuberculosis among people living with HIV. | To improve Tuberculosis screening among PLWH | A 3-antigen combination of Rv0934-P38, Ag85A, and Rv2031-HSPX outperformed the multi antigen panels used for TB screening among PLHIV, all three of them were significantly higher in people with TB compared to non-TB participants. |

| 2020 | Paul et al | 103 | Machine Learning Analysis Reveals Novel Neuroimaging and Clinical Signatures of Frailty in HIV | To predict the features that differentiate frail individuals from non-frail individuals using machine learning. | The linear machine learning model was able to distinguish frail from non-frail individuals based on a combination of lower CD4 count, poorer psychomotor performance, and various neuroimaging measures related to the visual and motor brain systems.  The interactive model identified novel synergies between neuroimaging features, female sex, symptoms of depression, and current CD4 count in predicting frailty risk. |
| --- | --- | --- | --- | --- | --- |
| 2020 | Rubin et al | 23 | Sex Differences in the Patterns and Predictors of Cognitive Function in HIV**.** | To use machine learning to identify profiles of cognitive functioning and their associated factors overall and within se | Sex was suggested as a main contributor in the heterogeneity in cognitive profiles in the two groups assessed and three cognitive profiles were identified overall and within each sex. the most discriminative factors were similar between men and women and included reading level (cognitive reserve), current and nadir CD4 count, plasma HIV viral load, duration of HIV disease, age, depressive symptoms, and race/ethnicity. |
| 2021 | Cardinal et al | 13 | Association between early carotid artery plaque presence, vascular strain imaging features and traditional cardiovascular risk factors in HIV infected individuals. | To identify associations between the presence of plaques and features consisting of traditional cardiovascular risk factors and strain elastography in normal sections of the common carotid artery | A machine learning method was able to discriminate between subjects with and without carotid artery plaques using a combination of traditional cardiovascular risk factors and strain elastography features, with reasonably good classification performance. The study identified associations between the presence of carotid artery plaques and the selected features in a population of HIV-positive and -negative individuals with low-to-intermediate cardiovascular risk. |
| 2021 | Tiburcio et al | 95 | Dynamics of T-Lymphocyte Activation Related to Paradoxical Tuberculosis-Associated Immune Reconstitution Inflammatory Syndrome in Persons With Advanced HIV. | To evaluate the patterns of T lymphocyte activation associated with TB-IRIS development following the start of ART in PLWH displaying high CD4+ T lymphocyte suppression pre-ART | phenotypic characterization of T cells:  TB-IRIS patients exhibited lower CD4+ and higher CD8+ T lymphocyte counts before ART in comparison to Non-IRIS individuals. TB-IRIS patients display heightened T lymphocyte activation with increased frequencies of proliferative CD4+ and cytotoxic CD8+ T cells during IRIS. D4+ T cell activation markers can serve as potential predictors of TB-IRIS, while the combination of CD4+ and CD8+ T cell markers is better at diagnosing TB-IRIS patients experiencing IRIS. |
| 2021 | Gelpi et al | 66 | The central role of the glutamate metabolism in long-term antiretroviral treated HIV-infected individuals with metabolic syndrome. | To investigate metabolic alterations in well‐treated PLWH with MetS to identify potential mechanisms behind the MetS phenotype | Altered amino acid metabolism, particularly glutamate metabolism, is a central characteristic of PLWH with MetS. A consensus approach identified 11 potential biomarkers of MetS in PLWH, 7 of which were upregulated in PLWH with MetS. A weighted co-expression network analysis showed that a single community of metabolites enriched in amino acids and peptides contained 6 of the 11 potential biomarkers, highlighting the central role of glutamate metabolism in MetS in PLWH. |
| 2021 | Yang et al | 6 | Utilizing electronic health record data to understand comorbidity burden among people living with HIV: a machine learning approach | To identify predictors of comorbidity burden among PLWH based on machine learning methods with electronic health record (EHR) data. | The outcome measured: change of comorbidity burden after HIV diagnosis: 28.2% of patients showed an increase of CCI score after HIV diagnosis. The top indicators for an increase in CCI score were: older age at HIV diagnosis, positive family history of chronic conditions, tobacco use, longer duration with retention in care, having PEBA insurance, having low recent CD4 count and duration of viral suppression. |
| 2021 | Shu et al | 24 | DNA methylation biomarker selected by an ensemble machine learning approach predicts mortality risk in an HIV-positive veteran population. | To identify the viral characteristics of HIV-1 subtype B tat that are associated with viral populations in the blood and CSF and neurocognitive dysfunction. | The nucleotide position (HXB2 position 5905) within the cysteine-rich domain appears to be a signature of the viral populations derived from the CSF HIV population diversity of that seems to be higher in subjects with HAND and a genetic between the blood and CSF derived that was not associated with neurocognitive impairment, it was inversely correlated with current and nadir CD4+ T cell counts. |
| 2021 | Yin et al | 98 | A Noninvasive Prediction Model for Hepatitis B Virus Disease in Patients with HIV: Based on the Population of Jiangsu, China. | To establish a machine learning model for identifying patients coinfected with hepatitis B virus (HBV) and human immunodeficiency virus (HIV) through two sexual transmission routes in Jiangsu, China | The univariate logistics regression combined with the AdaBoost algorithm could accurately screen the risk factors of HBV in HIV coinfection without invasive testing. AST was the most significant detective variable in both homosexual and heterosexual transmission groups |
| 2021 | Paul et al | 25 | Machine-learning classification of neurocognitive performance in children with perinatal HIV initiating de novo antiretroviral therapy. | To identify children with pHIV at risk for suboptimal neurocognitive development using a machine learning approach | Using HIV disease indices acquired from standard clinical measures can help distinguish children who are at risk for suboptimal neurocognitive development. Hematocrit levels from blood, and HIV disease dynamics (viral load and lymphocytes count) and mental health indices were identified as key predictor variables. |

| 2021 | Luckett et al | 26 | Modeling the Effects of HIV and Aging on Resting-State Networks Using Machine Learning. | To identify the RSN (resting state networks) that distinguishes controls from PLWH who were cognitively impaired and PLWH with no cognitive impairment. | new biomarkers of change in the organization of the brain were identified in this study.  PMN and SAL differentiated subjects according to their HIV status and FPN was associated with neurocognitive impairment  Anatomically differences in RSN topology were observed in the dorsal and rostral lateral prefrontal cortex, cingulate, and caudate |
| --- | --- | --- | --- | --- | --- |
| 2021 | Paul et al | 27 | Ensemble machine learning classification of daily living abilities among older people with HIV. | To identify novel factors that could help improve the diagnostic accuracy of impaired daily living abilities secondary to neurocognitive impairment (ADL) | Demographic and sociocultural factors, in addition to brain structure and function, were shown to be the main predictors for ADL. The need for more culturally relevant tests is needed to determine ADL status, especially for older PLWH. |
| 2021 | Solomon et al | 28 | White Matter Abnormalities Linked to Interferon, Stress Response, and Energy Metabolism Gene Expression Changes in Older HIV-Positive Patients on Antiretroviral Therapy. | To conduct a gene expression profiling on frontal white matter of PLWH on HAART and on negative controls and compare the two groups | a 15-gene signature predictive of HIV status was identified using machine learning and validated using results from prior studies. interferon response, stress response, energy metabolism, MHC-1, T cell, myeloid, and oligodendrocyte associated genes were implicated in HAND pathogenesis |
| 2021 | Zhao et al | 99 | Human immune deficiency virus-related structural alterations in the brain are dependent on age. | To determine if the brain alterations in PLWH are age-dependent using brain scans and machine learning algorithms | lower gray matter volumes (GMV), lower gyrification index, deeper sulcus depth, and larger cortical thickness (CTH) were observed in patients with HIV young-aged HIV patients displayed more severe morphological alterations than middle-aged HIV patients. They were also more vulnerable to HIV infection in brain structure when compared to middle-aged HIV patients. |
| 2021 | Finkelstein et al | 29 | Fixel-Based Analysis and Free Water Corrected DTI Evaluation of HIV-Associated Neurocognitive Disorders**.** | Using machine learning to compare between two novel techniques measuring White matter damage and determine their ability to distinguish between PLWH with and without neurocognitive impairment. | Machine learning classifiers using Fixel-based metrics (FBA) were better at distinguishing PLWH with neurocognitive impairment from the patients with normal cognition, better than free water corrected DTI metrics FBA can be considered a sensitive biomarker to monitor axonal degeneration in individuals with HIV infection. |
| 2021 | Oliveira et al | 30 | Longitudinal 5-year prediction of cognitive impairment among men with HIV disease. | To predict cognitive impairment(dementia) in the Multicenter AIDS Cohort Study participants and to determine the most influential factors that contribute to the prediction. | They accurately predicted neurocognitive impairment at an individual level although HIV infection was not a risk factor, clinical AIDS and Hepatitis B or C infection were identified as the strongest predictors of neurocognitive impairment. |
| 2021 | Pinheiro et al | 31 | An Intelligent Multicriteria Model for Diagnosing Dementia in People Infected with Human Immunodeficiency Virus. | Using a hybrid approach to accurately diagnose cognitive impairment in PLWH | The hybrid approach with the Machine Learning algorithm, Random Forest, combined with Verbal Decision Analysis, identified 10 characteristics that influenced the prediction of dementia in PLWH. |
| 2022 | Henderson et al | 16 | Predicting Risk of Multidrug-Resistant Enterobacterales Infections Among People With HIV. | To evaluate demographic and clinical predictors of MDR-E by estimating prevalence ratios (PRs) and employing machine learning classification algorithms | HIV-specific factors, including lower current and nadir CD4 cell counts, were strongly associated with having an MDR-E isolate. Established risk factors for MDR-E in the general population, such as recent hospitalization and certain comorbidities, were also associated with higher MDR-E prevalence among PWH. |
| 2022 | Villumsen et al | 67 | Integrative Lipidomics and Metabolomics for System-Level Understanding of the Metabolic Syndrome in Long-Term Treated HIV-Infected Individuals. | Comparison between PLWH without MetS and PLWH with MetS was conducted to identify a set of key lipids that define the mechanism of the lipid abnormalities of MetS in the context of HIV infection. | They denoted 13 differentially abundant lipids between PLWH without MetS and PLWH with MetS, which mainly belongs to diacylglyceride (DAG, n = 2) and triacylglyceride (TAG, n = 11) disruption of glutamate and fatty acid metabolism, suggesting their involvement in the pathogenesis of PLWH with MetS. Alterations in the lipid homeostasis and glutaminolysis need clinical interventions to prevent accelerated aging in PLWH with MetS. + exposure to earlier generations of antiretroviral therapy (ART) was associated with visceral adipose tissue (VAT) |
| 2022 | Womack et al | 32 | Predictive Risk Model for Serious Falls Among Older Persons Living With HIV | To predict serious falls in a group of middle aged PLWH who take ART | The model was more suitable to predict the risk of serious falls among an older population of PLWH  Including ART classes taken by these individuals did not improve the algorithm's prediction. |
| 2023 | Mohammadzadeh et al | 33 | The HIV Restriction Factor Profile in the Brain Is Associated with the Clinical Status and Viral Quantities. | To identify the expression profile of the Restriction factor in different categoried of HIV+ patients and classify them accordingly | RF (restriction factor) expression profile fluctuations are associated with the neurological status and the brain viral load and it ias also impacted by adherence to ART. Machine learning algorithms identified MAN1B1 as a key gene distinguishing between HIV [+] group from the HIV [+] groups with HAND. |

| 2023 | Murdoch et al | 34 | Neuroimaging and immunological features of neurocognitive function related to substance use in people with HIV. | To identify neuroimaging and immunological factors associated with substance use and that contribute to neurocognitive impairment (NCI) in people with HIV (PWH). | Using machine learning approach identified several biomarkers that contribute towards NCI prediction in PWH. These biomarkers were robust with respect to randomization of model hyperparameters, and to bootstrap resampling of participants |
| --- | --- | --- | --- | --- | --- |
| 2024 | Montesi et al | 64 | Predicting humoral responses to primary and booster SARS-CoV-2 mRNA vaccination in people living with HIV: a machine learning approach. | To develop a machine learning-based model able to predict the humoral response to mRNA vaccines in PLWH and to assess the impact of demographic and clinical variables on antibody production over time. | Non-linear machine learning algorithms, such as Random Forest, were more accurate than Generalized Linear Models in predicting humoral responses to SARS-CoV-2 mRNA vaccination in people living with HIV.  - Previous SARS-CoV-2 infection, BMI, CD4 T-cell count, and CD4/CD8 ratio were identified as the most influential factors in predicting primary cycle immunogenicity.  -The predictive value of these factors decreased after administration of booster vaccine doses. |
| 2024 | Niemczak et al | 84 | Machine learning for predicting cognitive deficits using auditory and demographic factors. | To determine if auditory variables combined with relevant demographic variables could predict cognitive deficits in PLWH better than demographic factors alone | Among the machine learning algorithms used, Gaussian naïve Bayes and Kernel Naïve Bayer has the highest accuracies when auditory variables were included in the model a combination of auditory variables and other variables affecting neurocognitive performance can be used to predict cognitive impairment. |
| 2021 | Menezes et al | 35 | Circulating plasma-derived extracellular vesicles expressing bone and kidney markers are associated with neurocognitive impairment in people living with HIV. | To determine if levels of extracellular vesicles from bone-and kidney-related cells associate with cognitive dysfunction | Machine learning model for identifying cognitive phenotypes in PWH on suppressive ART identified osteocalcin+EVs, nephrin+EVs, and CD24 + EVs as predictors cognitive impairment |

**Table S2.** Predicting drug resistance.

| **Year** | **Author** | **Ref.num** | **Title** | **Purpose** | **Findings** |
| --- | --- | --- | --- | --- | --- |
| 2002 | Beerenwinkel et al | 68 | Diversity and complexity of HIV-1 drug resistance: A bioinformatics approach to predicting phenotypes from genotype | To analyze correlations between HIV-1 genotype and resistance phenotype. | The decision tree algorithms were able to predict drug resistance phenotypes from HIV-1 genotypes with good accuracy, some key sequence positions that are associated with drug resistance were identified using a mutual information analysis. |
| 2006 | Rhee et al | 36 | Genotypic predictors of human immunodeficiency virus type 1 drug resistance | To relate HIV-1 protease and reverse transcriptase mutations to in vitro susceptibility to 16 antiretroviral drugs. | Least angle regression predicted susceptibility significantly better than other methods when using the complete set of mutations. |
| 2006 | Pillai et al | 63 | Genetic attributes of cerebrospinal fluid-derived HIV-1 env**.** | To analyze correlations between HIV-1 genotype and resistance phenotype | The decision tree algorithms were able to predict drug resistance phenotypes from HIV-1 genotypes with good accuracy, some key sequence positions that are associated with drug resistance were identified using a mutual information analysis. |
| 2009 | Wang et al | 69 | A comparison of three computational modelling methods for the prediction of virological response to combination HIV therapy | To develop neural network models able to predict virological response to therapy using HIV genotype and other clinical information. | The random forests (RF) algorithms had the highest correlation between predicted and actual virological response, followed by support vector machines (SVM) and then artificial neural networks (ANN). Combining the predictions of different s algorithms, such as ANN, RF, and SVM, can further improve the accuracy of predicting virological response to HIV treatment. |
| 2010 | Cheng and Carbonell | 62 | Automatic Detection of HIV Drug Resistance-Associated Mutations | To detect mutations associated with HIV drug -resistance without the need for expert knowledge. | Compared to the previous state-of-the-art with ample expert knowledge, fully automated prediction model for each drug yielded comparable performance at 82.9% classification accuracy and 0.819 coefficient of determination on average. |
| 2011 | Ravich et al | 37 | A combined sequence-structure approach for predicting resistance to the non-nucleoside HIV-1 reverse transcriptase inhibitor Nevirapine. | To develop a combined sequence–structure approach to predicting NVP drug resistance based on a patient's mutational patterns in the target protein. | SVM and RF were applied to a subset of RT mutants with an established resistance or sensitivity to Nevirapine. Both demonstrated high accuracy predicting susceptibility to Nevirapine. |
| 2012 | Zazzi et al | 70 | Predicting Response to Antiretroviral Treatment by Machine Learning: The EuResist Project | To learn from the large data sets of patient histories and can provide an objective and accurate estimate of the virological response to different antiretroviral regimens. | All 3 engines have a similar performance but are not identical and in fact disagree in 18.3% of cases. These engines may valuably assist the HIV specialist in establishing effective regimens for patients with drug-resistant virus strains. |
| 2012 | Masso | 38 | Prediction of human immunodeficiency virus type 1 drug resistance: Representation of target sequence mutational patterns via an n-grams approach | To develop predictive models of HIV-1 drug resistance based on mutational patterns in the protease (PR) and reverse transcriptase (RT) enzymes. | Predictive models of drug resistance for HIV-1 protease (PR) and reverse transcriptase (RT) inhibitors based on mutational patterns in the target sequences were developed.Their performance was evaluated using cross-validation and two different approaches for representing the mutant protein. sequences as feature vectors were compared. |
| 2013 | Heider et al | 71 | Multilabel classification for exploiting cross-resistance information in HIV-1 drug resistance prediction | To provide regarding the statistical dependencies between HIV-1 drugs is instrumental in learning classifiers for resistance prediction | Cross-resistance information can be used to improve overall accuracy in drug resistance prediction. By using MLC methods (especially CC) they were able to exploit cross-resistance information for RT inhibitors. The study only focused on nucleoside analogues (NRTI). |
| 2013 | Yu et al | 39 | Prediction of HIV drug resistance from genotype with encoded three-dimensional protein structure. | To improve the computational prediction of resistance from genotype data. | SVM performance (accuracy and sensitivity) showed that the novel encoding using Delaunay triangulation separates the resistant and non-resistant data into two distinct categories. Classification with the structural encoding significantly outperforms other state of the art methods (HIV-GRADE, ANRS-rules, Stanford HIVdb, and Rega) for predicting resistance to PIs, NRTI and NNRTI for the three tested classifiers SVM, ANN and the sparse dictionary. |
| 2016 | Shen et al | 40 | Automated prediction of HIV drug resistance from genotype data. | To predict phenotypic resistance from genotype data of HIV protease (PR) and reverse transcriptase (RT) using a unified encoding of protein sequence and structure. | Highly accurate prediction of drug resistance from genotype data of HIV protease (PR) and reverse transcriptase (RT) using classification with RF and KNN (using a unified encoding of sequence and protein structure as a feature vector = compact and efficient) |
| 2016 | Riemenschneider et al | 72 | Exploiting HIV-1 protease and reverse transcriptase cross-resistance information for improved drug resistance prediction by means of multi-label classification | Using a multi-label classification model incorporating cross-resistance information to predict drug resistance for two of the major drug classes used in antiretroviral therapy for HIV-1. | MLC model improved the accuracy of predicting drug resistance for protease inhibitors (PIs) and non-nucleoside reverse transcriptase inhibitors (NNRTIs) compared to previous binary classification models. for NNRTIs, the strongest association can be observed between NVP and EFV. for PI: RTV and IDV having the strongest correlation. |
| 2016 | Riemenschneider et al | 73 | SHIVA - a web application for drug resistance and tropism testing in HIV. | To develop a web service for drug resistance prediction of commonly used drugs in antiretroviral therapy. | The algorithm can handle several million sequences (more than the available online services) SHIVA provides drug resistance models for 23 drugs (PIs, NRTIs, NNRTIs, INIs, and BVM) |
| 2017 | Singh | 85 | Machine Learning to Improve the Effectiveness of ANRS in Predicting HIV Drug Resistance | To improve the prediction of the ANRS gold standard in predicting HIV drug resistance | The ANRS algorithm was improved, other mutations that do not exist in the ANRS rules were found to contribute to resistance for all 10 ARVs tested |
| 2018 | Pawar et al | 41 | Analysis of drug resistance in HIV protease. | To predict drug resistance profiles from genomic data using a classification machine. | RBM combined with structure-based encoding are well-suited to the prediction of drug resistance in HIV PR. The graph-based structure/sequence encoding used removes much of the redundancy in the data. |
| 2019 | Ramon et al | 74 | HIV drug resistance prediction with weighted categorical kernel functions. | Using weighted categorical kernel functions to predict drug resistance from virus sequence data. And compare it to non-categorical kernels like RF and RBF | Using kernel functions that directly address the presence of allele mixtures, and the categorical nature of the data is a novel approach to predict drug resistance in HIV The Jaccard kernel was the best method, either in its weighted or unweighted form, for 20 out of the 21 drugs and weighted Jaccard outperforms ANN in all drugs |
| 2020 | Steiner et al | 42 | Drug Resistance Prediction Using Deep Learning Techniques on HIV-1 Sequence Data. | To apply interpretable deep learning techniques to classify amino acid sequences by drug resistance phenotypes. And to compare those ML architectures while addressing model interpretability and its biological implications. | Convolutional neural networks are the best performing architecture and display a correspondence between the importance of biologically relevant features in the classifier and overall performance. Average performance was best for PI Data set then NRTI and then NNRTI. |
| 2021 | Ekpenyong et al | 86 | A transfer learning approach to drug resistance classification in mixed HIV dataset | To classify patients’ response to failed treatments due to adverse drug reactions to ensure an early detection of patients' response to treatments. | The DNN architecture yielding best performance measures, when compared to state-of-the-art methods, transfer learning has the best classification accuracy, but its recall rate is slightly lower than Fuzzy-multidimensional deep learning. |
| 2021 | Blassel et al | 104 | Using machine learning and big data to explore the drug resistance landscape in HIV. | To discriminate RTI-naive from RTI-experienced sequences. Without the need for phenotypic data using larger Data. | Six mutations that might play a role in drug resistance in HIV were identified from classifiers. Logistic regression showed similar or better performance than other classifiers with easier interpretation regarding the nature or resistance level of a given sequence. |
| 2022 | Dampier et al | 43 | HIV- Bidirectional Encoder Representations From Transformers: A Set of Pretrained Transformers for Accelerating HIV Deep Learning Tasks | To investigate the ability of BERT to predict HIV-1- related phenotypes including Protease Drug Resistance, coreceptor usage and body site identification. | Bidirectional Encoder Representations from Transformers (BERT), a protein-based transformer model fine-tuned on HIV-1 genomic sequences, was able to achieve high accuracies on protease inhibitor resistance, coreceptor utilization, and body-site identification respectively. |
| 2023 | Tunc et al | 75 | Machine learning aided multiscale modelling of the HIV-1 infection in the presence of NRTI therapy | To address the impact of existing mutations, timing of initiation, and adherence levels of nucleotide reverse transcriptase inhibitors (NRTIs) on the evolutionary dynamics of the virus strains using a mathematical approach and a within host model of HIV infection. | Based on their mathematical model and simulation: D4T-3TC, D4T-AZT, and TDF-D4T combinations are less likely to result in treatment failure due to their pharmacokinetic parameters and IC50 values in the presence of various viral strains. Undetected viral strains at the diagnosis as well as the time of initiation of therapy may have considerable effects on treatment efficacy. |
| 2023 | Tunc et al | 76 | Prediction of HIV-1 protease resistance using genotypic, phenotypic, and molecular information with artificial neural networks | To construct drug isolate fold (DIF) change-based artificial neural network (ANN) models for estimating the resistance potential of molecules inhibiting the HIV-1 protease (PR) enzyme. | The machine learning model constructed can simultaneously take molecular fingerprints and mutational information jointly as inputs to estimate the fold change values and it accurately predicted the drug resistance tendencies of each inhibitor pair. |
| 2023 | Paremskaia et al | 77 | Web Service for HIV Drug Resistance Prediction Based on Analysis of Amino Acid Substitutions in Main Drug Targets | To predict HIV drug resistance related to amino acid substitutions in HIV targets using appropriate machine learning methods. | Classification methods have relatively high reliability and were used to create a web application, HVR (HIV drug Resistance), for the prediction of HIV drug resistance to PI and RT inhibitors based on the analysis of the amino acid sequences of the appropriate HIV proteins from clinical samples. |

**Table S3.** Monitoring HIV/AIDS infection

| **Year** | **Author** | **Ref.num** | **Title** | **Purpose** | **Findings** |
| --- | --- | --- | --- | --- | --- |
| 2005 | Pillai et al | 44 | Semen-specific genetic characteristics of human immunodeficiency virus type 1 env | To determine if HIV in semen has a different signature compared to the blood's HIV | Machine learning algorithms could predict seminal tropism based on env sequences with accuracies exceeding 90%. Semen-derived HIV-1 viral populations exhibit less diversity, lower levels of positive selection, decreased CXCR4 coreceptor usage, and altered glycosylation patterns compared to blood-derived virus. |
| 2017 | Rojas Sanchez et al | 78 | Impact of Clinical Parameters in the Intrahost Evolution of HIV-1 Subtype B in Pediatric Patients: A Machine Learning Approach | Using machine learning approach to determine the impact of several clinical factors on the intrahost evolution of HIV-1 subtype B. | Six supervised classification methods to predict three HIV-1B evolutionary parameters (diversity (d), nonsynonymous and synonymous mutations (dN, dS)) and the fixation of drug resistance mutations (DRM)  the best predictive variables for HIV-1B evolutionary parameters were the age of HIV-1 diagnosis ford, the age at first ART for dN and the year of HIV-1 diagnosis ds. The year of infection and year of sampling were relevant for the fixation of drug resistance mutations, including those specific to protease inhibitors. |
| 2019 | Ahlström et al | 79 | Algorithmic prediction of HIV status using nation-wide electronic registry data | To predict HIV status using electronic health records (EHR) | All machine learning algorithms applied in this study can accurately identify undiagnosed PLWH using data from EHR. If integrated into clinical software, it could identify people suitable for treatment and improve early diagnosis of HIV for pre -exposure prophylaxis (PrEP) |
| 2019 | Pohlmeyer et al | 45 | Identification of NK Cell Subpopulations That Differentiate HIV-Infected Subject Cohorts with Diverse Levels of Virus Control | To identify new NK cells phenotypes and subpopulations that are associated with immunological control on HIV infection using different approaches | A subpopulation of CD56dim CD16+ NK cells (known as CD11b+ CD57− CD161+ Siglec-7+) was identified and it distinguishes HIV controllers from non-controllers as it is more abundantly found in HIV elite controllers compared to viremic non-controllers. |
| 2020 | Kimaina et al | 87 | Comparison of machine learning methods for predicting viral failure: a case study using electronic health record data. | To identify patients at risk of HIV viral at the first and second measurements following initiation of antiretroviral therapy | Ensemble machine learning techniques, particularly Super Learner, gradient boosting, and Bayesian additive regression trees, outperformed other methods in predicting viral failure at both the first and second follow-up measurements after initiation of antiretroviral therapy. |
| 2021 | Weissman et al | 46 | Using a machine learning approach to explore predictors of healthcare visits as missed opportunities for HIV diagnosis | Using a machine learning approach to identify the predictors for the missed opportunities for earlier HIV diagnosis using electronic health records data (EHR). | This study identified factors such as visiting the emergency department, being older, being male, and having alcohol use were associated with missed opportunities for earlier HIV diagnosis. Implementing an improved HIV testing at the emergency department can improve early HIV diagnosis. |
| 2021 | Giron et al | 47 | Non-invasive plasma glycomic and metabolic biomarkers of post-treatment control of HIV | To identify metabolic and glycomic biomarkers of viral rebound in patients from two independent cohort’s pre-treatment interruption | Plasma glycomic and metabolic biomarkers that can predict the duration and probability of HIV remission pre-ATI were identified in this study.  Using machine learning algorithms with a combination of those biomarkers are able to predict time to viral rebound and the probability of viral remission with high accuracy. |
| 2021 | Murnane et al | 48 | Machine Learning Algorithms Using Routinely Collected Data Do Not Adequately Predict Viremia to Inform Targeted Services in Postpartum Women Living With HIV | To predict viremia to facilitate targeted adherence support in sub-Saharan Africa during postpartum. | The machine learning model developed to identify women most likely to experience viremia during post-partum achieved moderate model discrimination but insufficient prediction performance. Using routinely collected data is not enough to adequately predict viremia in postpartum women living with HIV to inform targeted adherence support services. |
| 2022 | Li et al | 100 | Construction of Machine Learning Models to Predict Changes in Immune Function Using Clinical Monitoring Indices in HIV/AIDS Patients After 9.9-Years of Antiretroviral Therapy in Yunnan, China | To use machine learning models and incorporate clinical indicators to predict changes in immune function. | The three machine learning methods (SVM, RF, and MLP) had better predictive capability for the CD4/CD8 ratio in patients with baseline CD4 counts ≥200 cells/ml compared to those with <200 cells/ml.  The SVM algorithm performed best for predicting CD4/CD8 ratio in patients with baseline CD4 counts <200 cells/ml.  The RF algorithm performed best for predicting CD4/CD8 ratio in patients with baseline CD4 counts ≥200 cells/ml. |
| 2022 | Ragonnet-Cronin et al | 105 | Human Immunodeficiency Virus (HIV) Genetic Diversity Informs Stage of HIV-1 Infection Among Patients Receiving Antiretroviral Therapy in Botswana. | Using machine learning algorithms to identify the HIV infection stage based on the viral sequences | A machine learning method was optimized to classify infections as less than or greater than 1 year old based on viral genetic diversity, demographic, and clinical data and it was tested on data from newly diagnosed participants, both with and without documented negative HIV tests |
| 2022 | Prosperi et al | 50 | Identification of Social and Racial Disparities in Risk of HIV Infection in Florida using Causal AI Methods | To identify the social and racial disparities that contribute to the risk of HIV infection among people in Florida | The study found that The risk of HIV infection was higher for African Americans compared to non-African Americans,  Using the FACTS method helped identifying several social determinants of health (SDoH), including education, income, violent crime, drinking, smoking, and rurality, that contribute to The racial disparity in HIV risk. |
| 2023 | Kagendi and Mwau | 88 | A Machine Learning Approach to Predict HIV Viral Load Hotspots in Kenya Using Real-World Data. | To develop a suitable machine learning model able to accurately predict viral load VL hotspots in Kenya an establish an early warning system | The random forest algorithm developed accurately classified VL hotspots in Kenya thus identifying the regions that need the most attention (1 month ahead in time) as well as predicting 434 additional new VL hotspots |
| 2024 | Mahto and Sood | 49 | HIV Progression and Outcome Prediction to Enhance Patient Matching for Clinical Trials | Using machine learning to predict HIV progression to enhance patient matching for clinical trials. | The study shows the potential of machine learning to accurately classify individuals with HIV into distinct outcome categories.  Random Forest and XGBoost algorithms achieved the highest accuracy in predicting HIV progression. |

**Table S4.** Predicting treatment outcomes.

| **Year** | **Author** | **Ref.num** | **Title** | **Purpose** | **Findings** |
| --- | --- | --- | --- | --- | --- |
| 2023 | Mamo et al | 89 | Machine learning to predict virological failure among HIV patients on antiretroviral therapy in the University of Gondar Comprehensive and Specialized Hospital, in Amhara Region, Ethiopia, 2022 | To use machine learning methods to predict the features that cause virological failure in HIV-positive patients receiving antiretroviral therapy. | Amongst the 7 classifiers, random forest has the best performance predicting virological failure, it also identified the top eight predictors (Male, younger age, longer duration on ART, not taking CPT, not taking TPT, secondary educational status, TDF-3TC-EFV, and low CD4 counts) of virological failure. CD4 count was the most important predictor. |
| 2018 | Bisaso et al | 90 | A comparative study of logistic regression-based machine learning techniques for prediction of early virological suppression in antiretroviral initiating HIV patients | To employ machine learning models to predict virologic suppression in patients based on clinical and demographic variables. | MTLR has the best predictive performance regarding virological suppression while using readily available demographic and clinical variables and could be used to derive a risk score for use in resource-limited settings. |
| 2010 | Bogojeska et al | 80 | Dealing with sparse data in predicting outcomes of HIV combination therapies | To predict virological response to antiretroviral therapy using a logistic regression model for each combination of therapy. | The model is trained to be able to integrate phenotypic information on therapy outcomes and provides information on the in vivo effectiveness of each drug and it’s not only advantageous for therapies with few training samples, but also for all other therapies. |
| 2011 | Zazzi et al | 81 | Prediction of response to antiretroviral therapy by human experts and by the EuResist data-driven expert system (the EVE study) | To develop a data driven expert system able to predict the response to antiretroviral therapy. | The EuResist expert system performed at least as well as 10 human HIV drug resistance experts in predicting short-term virological response to antiretroviral therapy. |
| 2017 | Kebede et al | 91 | Predicting CD4 count changes among patients on antiretroviral treatment: Application of data mining techniques | To use machine learning algorithms to predict CD 4 counts changes patients taking ART, using their electronic records. | Random forest has the high accuracy predicting CD4 count changes with a precision and sensitivity and recall values neighboring 99%. |
| 2019 | Ekpenyong et al | 92 | Fuzzy-multidimensional deep learning for efficient prediction of patient response to antiretroviral therapy | To predict the patients' response to ART using machine learning and multidimensional scaling. | Clustering results: immunological and RNA changes were observed in the Akwa-Ibom HIV database compared to the Stanford HIV database, before and after ART.  The Levenberg-Marquardt algorithm for deep neural networks, combined with multidimensional scaling, provided the best performance in predicting patient response to antiretroviral therapy. |
| 2019 | Lee et al | 51 | A comparison of machine learning techniques for classification of HIV patients with antiretroviral therapy-induced mitochondrial toxicity from those without mitochondrial toxicity | To determine if the levels of ribonucleotides (RN) and dNTP pool sizes can be used to distinguish between PLWH with mitochondrial toxicity and PLWH without toxicity. | Changes in RN and dNTP pools in participants with mitochondrial were observed and they can be considered as biomarkers ART-induced mitochondrial toxicity. Complex tree-based machine learning methods performed best for classifying patients based on dNTP (classification tree and Ada boost) and RN (random forest) levels, but simpler methods like Linear Discriminant Analysis and Logistic Regression also performed well. |
| 2021 | Bose et al | 52 | Minimum redundancy maximal relevance gene selection of apoptosis pathway genes in peripheral blood mononuclear cells of HIV-infected patients with antiretroviral therapy-associated mitochondrial toxicity | using the mRMR algorithm to rank the most relevant and least redundant genes and to test the prediction accuracy of the differently expressed genes. | MRMR algorithms were applied on the differentially expressed genes between the cases and controls and ranked two proapoptotic genes (DFFA and TNFRSF1A) on top. The network analysis revealed distinct gene interaction patterns between the case and control groups. |
| 2022 | Streeck et al | 106 | Dissecting drivers of immune activation in chronic HIV-1 infection | To identify markers of immune activation within the AFRICOS cohort. | Markers of immune activations in PLWH were different from those of people without HIV. Among PLWH with detectable viral loads, most immune parameters are significantly correlated with viral load, except for IFN-α. Different inflammatory profiles were different when comparing men and women living with HIV and individuals off ART and with HIV viremia. |
| 2023 | Mulyadi and Qomariyah | 101 | Using Machine Learning to Analyse the Effect of Antiretroviral therapy (ART) on People with HIV | To analyze the effects of antiretroviral therapy on PLWH using different machine learning algorithms. | Amongst the 7 classifiers, random forest has the best performance predicting virological failure, it also identified the top eight predictors (Male, younger age, longer duration on ART, not taking CPT, not taking TPT, secondary educational status, TDF-3TC-EFV, and low CD4 counts) of virological failure. CD4 count was the most important predictor. |
| 2024 | Premeaux et al | 53 | Machine learning models based on fluid immunoproteins that predict non-AIDS adverse events in people with HIV | To generate a machine learning model able to classify PLWH who experienced NAE based on demographic and clinical data. | The SVM algorithm can predict non-fatal NAEs among PWH with high accuracy pre-ART initiation and one-year post-ART at viral suppression. |

**Table S5.** Treatment adherence.

| **Year** | **Author** | **Ref.num** | **Title** | **Purpose** | **Findings** |
| --- | --- | --- | --- | --- | --- |
| 2021 | Oliwa et al | 54 | Development of a predictive model for retention in HIV care using natural language processing of clinical notes. | To predict retention in HIV care using natural language processing of clinical notes of patients | The predictive model has a strong performance. It could help identify patients at risk of falling out of HIV care. Characteristics associated with being lost to follow-up included mentions of MSM status, HCV, substance abuse, and unemployment, while characteristics associated with retention in care included mentions of being "well on ART", pregnancy, and congenital HIV. |
| 2020 | Ramachandran et al | 55 | Predictive Analytics for Retention in Care in an Urban HIV Clinic. | To identify and classify patients who are at risk of dropping out of care | The machine learning model developed identified patients at risk of dropping out of HIV care and has the potential to improve retention in care. The most important predictor variables were based on previous retention history and clinic visit history. |
| 2020 | Benitez et al | 107 | Super learner analysis of real-time electronically monitored adherence to antiretroviral therapy under constrained optimization and comparison to non-differentiated care approaches for persons living with HIV in rural Uganda. | To develop a machine learning model using EAM data in order to predict the risk of viremia in PLWH in rural Uganda | The real-time EAM-based machine learning model predicted effectively viremia in PLWH it can also be used to guide viral load testing in order to reduce the number of viral load tests ordered. |
| 2022 | Maskew et al | 93 | Applying machine learning and predictive modeling to retention and viral suppression in South African HIV treatment cohort. | To identify patients at risk for loss-of-follow up and predict viremial using machine learning methods | The machine learning models developed can be used to identify HIV patients at risk of disengaging from care or having unsuppressed viral loads, which can then inform targeted interventions to improve retention and viral suppression. |
| 2015 | Petersen et al | 56 | Super Learner Analysis of Electronic Adherence Data Improves Viral Prediction and May Provide Strategies for Selective HIV RNA Monitoring | To use machine learning algorithms to analyze electronic adherence data from HIV patients on antiretroviral therapy. | Applying Super Learner analysis to electronic medication adherence data, CD4 count, and ART regimen data significantly improved prediction of virological failure compared to using a single adherence measure alone.  This model could allow 25-31% of viral load tests to be avoided while still detecting 95% of virological failures, leading to cost savings of $16-$29 per person-month. |
| 2023 | Ogbechie et al | 94 | Predicting Treatment Interruption Among People Living With HIV in Nigeria: Machine Learning Approach. | To predict interruption in treatment (IIT) at 30 days among people living with HIV newly enrolled on ART in Nigeria using machine learning methods. | The model developed used routinely collected individual- and clinic-level data, and it has a high predictive value. And has been incorporated into the national systems for routine individual-level case management and monitoring and evaluation in pilot clinics. |
| 2022 | Stockman et al | 108 | Predictive Analytics Using Machine Learning to Identify ART Clients at Health System Level at Greatest Risk of Treatment Interruption in Mozambique and Nigeria**.** | Using machine learning methods to identify patients who are at risk of falling out of care | The machine learning models developed showed strong predictive performance in identifying ART clients at greatest risk of treatment interruption in both Mozambique and Nigeria. Therefore, it can help prioritize clients for follow-up and interventions accordingly. |
| 2005 | Lu et al | 57 | Multi-class support vector machines for modeling HIV/AIDS treatment adherence using patient data. | To identify the variables that are predictive of patient’s adherence to ART to classify those patients' adherence according to those factors they predict new patients' adherence using the previous classification | 9 predictive variables were identified  SVM performed better than NN when quantitatively modeling the relationship between patient factors and HIV/AIDS treatment adherence, |

**Table S6.** Treatment recommendations.

| **Year** | **Author** | **Ref.num** | **Title** | **Purpose** | **Findings** |
| --- | --- | --- | --- | --- | --- |
| 2005 | Ying et al | 58 | A fuzzy discrete event system with self-learning capability for HIV/AIDS treatment regimen selection**.** | To investigate the ability of a self-learning system to select appropriate ART regimen | The fuzzy discrete event system developed had high self-learning accuracy when comparing actual treatments taken by patients and those suggested by the new system. Compared to neural networks, the system was more transparent and interpretable, it can be used to quantitatively evaluate the clinical utility of HIV/AIDS treatment regimens, even before they are available, with minimal involvement of expert clinicians |
| 2006 | Ying et al | 59 | A fuzzy discrete event system approach to determining optimal HIV/AIDS treatment regimens. | To develop a self-learning system that can provide accurate treatment regimen selection for PLWH | The authors developed an EFDES-based HIV/AIDS regimen selection system that can handle higher levels of uncertainty compared to the previous FDES-based system.  - The EFDES-based system matched the actual prescribed regimens for 35 patients over 80% of the time, which is about the same as the FDES  -based system but under more realistic and practical conditions. |
| 2007 | Ying et al | 60 | A self-learning fuzzy discrete event system for HIV/AIDS treatment regimen selection. | To develop a self-learning system able to select HIV treatment regimen as well as an physician (expert or non-expert) | The self-learning HIV/AIDS regimen selection system was able to accurately predict the suitability of new treatment regimens with an accuracy between 84.4% and 100%.  - The system's treatment regimen selections agreed with those of experienced non-expert physicians 82.9% of the time and agreed 100% with the selections of expert physicians. |
| 2008 | Rosen-Zvi et al | 82 | Selecting anti-HIV therapies based on a variety of genomic and clinical factors | To determine if combining genotypic, clinical and demographic factors improves prediction of anti-HIV therapy compared to using genotypic information alone. | A combination of the three engines taking into consideration all factors (clinical, demographic and genotypic information) have the best prediction performance. It provides a recommendation for HAART therapy, given the genotype |
| 2015 | Herrera-Ibatá et al | 109 | Mapping chemical structure-activity information of HAART-drug cocktails over complex networks of AIDS epidemiology and socioeconomic data of US counties | Using machine learning models to predict the best HAART combinations based on several factors | Linear neural network model that could predict HAART cocktails with good performance and with multilayer perceptron model, the researchers were able to achieve even higher performance. They were also able to  predict if drugs in preclinical assays were effective for the treatment of HIV in different populations of U.S. counties with a given AIDS epidemiological prevalence. |
| 2024 | Pham et al | 183 | DeepARV: ensemble deep learning to predict drug-drug interaction of clinical relevance with antiretroviral therapy. | To assess the potential of deep learning approaches to predict DDIs of clinical relevance between ARVs and comedications. | DeepARV-Sim and DeepARV-ChemBERTa deep learning algoritms were able to predict clinically relevant drug-drug interactions between antiretroviral drugs and comedications with high accuracy. The algorithms leveraged drug molecular structure information and addressed class imbalance in the data to improve their predictive ability on clinically relevant DDIs. |

**References**

6.  Yang X, Zhang J, Chen S, Weissman S, Olatosi B, Li X. Utilizing electronic health record data to understand comorbidity burden among people living with HIV: a machine learning approach. AIDS. May 1, 2021;35(Suppl 1):S39-S51. [doi: ] [Medline: 33867488]

13.  Cardinal MHR, Durand M, Chartrand-Lefebvre C, Soulez G, Tremblay C, Cloutier G. Association between early carotid artery plaque presence, vascular strain imaging features and traditional cardiovascular risk factors in HIV infected individuals. Presented at: 2021 IEEE International Ultrasonics Symposium (IUS); Sep 11-16, 2021:1-3; Xi’an, China. [doi: ]

14.  Kozak I, Sample PA, Hao J, et al. Machine learning classifiers detect subtle field defects in eyes of HIV individuals. Trans Am Ophthalmol Soc. 2007;105:111-118. [Medline: 18427600]

15.  Goldbaum MH, Kozak I, Hao J, et al. Pattern recognition can detect subtle field defects in eyes of HIV individuals without retinitis under HAART. Graefes Arch Clin Exp Ophthalmol. Apr 2011;249(4):491-498. [doi: ] [Medline: 20865422]

16.  Henderson HI, Napravnik S, Kosorok MR, et al. Predicting risk of multidrug-resistant enterobacterales infections among people with HIV. Open Forum Infect Dis. Oct 2022;9(10):ofac487. [doi: ] [Medline: 36225740]

17.  Holman AG, Gabuzda D. A machine learning approach for identifying amino acid signatures in the HIV env gene predictive of dementia. PLoS ONE. 2012;7(11):e49538. [doi: ] [Medline: 23166702]

18.  Choi JY, Hightower GK, Wong JK, et al. Genetic features of cerebrospinal fluid-derived subtype B HIV-1 tat. J Neurovirol. Apr 2012;18(2):81-90. [doi: ] [Medline: 22528397]

19.  Cassol E, Misra V, Dutta A, Morgello S, Gabuzda D. Cerebrospinal fluid metabolomics reveals altered waste clearance and accelerated aging in HIV patients with neurocognitive impairment. AIDS. Jul 17, 2014;28(11):1579-1591. [doi: ] [Medline: 24752083]

20.  Underwood J, Cole JH, Leech R, Sharp DJ, Winston A, CHARTER group. Multivariate pattern analysis of volumetric neuroimaging data and its relationship with cognitive function in treated HIV disease. J Acquir Immune Defic Syndr. Aug 1, 2018;78(4):429-436. [doi: ] [Medline: 29608444]

21.  Luckett P, Paul RH, Navid J, et al. Deep learning analysis of cerebral blood flow to identify cognitive impairment and frailty in persons living with HIV. J Acquir Immune Defic Syndr. Dec 15, 2019;82(5):496-502. [doi: ] [Medline: 31714429]

22.  Dastgheyb RM, Sacktor N, Franklin D, et al. Cognitive trajectory phenotypes in human immunodeficiency virus–infected patients. J Acquir Immune Defic Syndr. Sep 1, 2019;82(1):61-70. [doi: ] [Medline: 31107302]

23.  Rubin LH, Sundermann EE, Dastgheyb R, et al. Sex differences in the patterns and predictors of cognitive function in HIV. Front Neurol. 2020;11:551921. [doi: ] [Medline: 33329301]

24.  Shu C, Justice AC, Zhang X, et al. DNA methylation biomarker selected by an ensemble machine learning approach predicts mortality risk in an HIV-positive veteran population. Epigenetics. 2021;16(7):741-753. [doi: ] [Medline: 33092459]

25.  Paul RH, Cho KS, Belden AC, et al. Machine-learning classification of neurocognitive performance in children with perinatal HIV initiating de novo antiretroviral therapy. AIDS. Apr 1, 2020;34(5):737-748. [doi: ] [Medline: 31895148]

26.  Luckett PH, Paul RH, Hannon K, et al. Modeling the effects of HIV and aging on resting-state networks using machine learning. J Acquir Immune Defic Syndr. Dec 1, 2021;88(4):414-419. [doi: ] [Medline: 34406983]

27.  Paul R, Tsuei T, Cho K, et al. Ensemble machine learning classification of daily living abilities among older people with HIV. EClinicalMedicine. May 2021;35:100845. [doi: ] [Medline: 34027327]

28.  Solomon IH, Chettimada S, Misra V, et al. White matter abnormalities linked to interferon, stress response, and energy metabolism gene expression changes in older HIV-positive patients on antiretroviral therapy. Mol Neurobiol. Feb 2020;57(2):1115-1130. [doi: ] [Medline: 31691183]

29.  Finkelstein A, Faiyaz A, Weber MT, et al. Fixel-based analysis and free water corrected DTI evaluation of HIV-associated neurocognitive disorders. Front Neurol. 2021;12:725059. [doi: ] [Medline: 34803875]

30.  Oliveira NL, Kennedy EH, Tibshirani R, et al. Longitudinal 5-year prediction of cognitive impairment among men with HIV disease. AIDS. May 1, 2021;35(6):889-898. [doi: ] [Medline: 33534203]

31.  Pinheiro L, Pereira MLD, Andrade E de, et al. An intelligent multicriteria model for diagnosing dementia in people infected with human immunodeficiency virus. Appl Sci (Basel). 2021;11(21):10457. [doi: ]

32.  Womack JA, Murphy TE, Leo-Summers L, et al. Predictive risk model for serious falls among older persons living with HIV. J Acquir Immune Defic Syndr. Oct 1, 2022;91(2):168-174. [doi: ] [Medline: 36094483]

33.  Mohammadzadeh N, Zhang N, Branton WG, et al. The HIV restriction factor profile in the brain is associated with the clinical status and viral quantities. Viruses. Jan 23, 2023;15(2):316. [doi: ] [Medline: 36851531]

34.  Murdoch DM, Barfield R, Chan C, et al. Neuroimaging and immunological features of neurocognitive function related to substance use in people with HIV. J Neurovirol. Feb 2023;29(1):78-93. [doi: ] [Medline: 36348233]

35.  Marques de Menezes EG, Bowler SA, Shikuma CM, Ndhlovu LC, Norris PJ. Circulating plasma-derived extracellular vesicles expressing bone and kidney markers are associated with neurocognitive impairment in people living with HIV. Front Neurol. 2024;15:1383227. [doi: ] [Medline: 38725641]

36.  Rhee SY, Taylor J, Wadhera G, Ben-Hur A, Brutlag DL, Shafer RW. Genotypic predictors of human immunodeficiency virus type 1 drug resistance. Proc Natl Acad Sci U S A. Nov 14, 2006;103(46):17355-17360. [doi: ] [Medline: 17065321]

37.  Ravich VL, Masso M, Vaisman II. A combined sequence-structure approach for predicting resistance to the non-nucleoside HIV-1 reverse transcriptase inhibitor Nevirapine. Biophys Chem. Jan 2011;153(2-3):168-172. [doi: ] [Medline: 21146283]

38.  Masso M. Prediction of human immunodeficiency virus type 1 drug resistance: representation of target sequence mutational patterns via an n-grams approach. Presented at: 2012 IEEE International Conference on Bioinformatics and Biomedicine; Oct 4-7, 2012:1-6; Philadelphia, PA, USA. 2012.[doi: ]

39.  Yu X, Weber IT, Harrison RW. Prediction of HIV drug resistance from genotype with encoded three-dimensional protein structure. BMC Genomics. 2014;15 Suppl 5(Suppl 5):1-13. [doi: ] [Medline: 25081370]

40.  Shen C, Yu X, Harrison RW, Weber IT. Automated prediction of HIV drug resistance from genotype data. BMC Bioinformatics. Aug 31, 2016;17 Suppl 8(Suppl 8):278. [doi: ] [Medline: 27586700]

41.  Pawar SD, Freas C, Weber IT, Harrison RW. Analysis of drug resistance in HIV protease. BMC Bioinformatics. Oct 22, 2018;19(Suppl 11):362. [doi: ] [Medline: 30343664]

42.  Steiner MC, Gibson KM, Crandall KA. Drug resistance prediction using deep learning techniques on HIV-1 sequence data. Viruses. May 19, 2020;12(5):560. [doi: ] [Medline: 32438586]

43.  Dampier W, Link RW, Earl JP, et al. HIV- bidirectional encoder representations from transformers: a set of pretrained transformers for accelerating HIV deep learning tasks. FrontVirol. 2022;2:880618. [doi: ]

44.  Pillai SK, Good B, Pond SK, et al. Semen-specific genetic characteristics of human immunodeficiency virus type 1 env. J Virol. Feb 2005;79(3):1734-1742. [doi: ] [Medline: 15650198]

45.  Pohlmeyer CW, Gonzalez VD, Irrinki A, et al. Identification of NK cell subpopulations that differentiate HIV-infected subject cohorts with diverse levels of virus control. J Virol. Apr 1, 2019;93(7):10-1128. [doi: ] [Medline: 30700608]

46.  Weissman S, Yang X, Zhang J, Chen S, Olatosi B, Li X. Using a machine learning approach to explore predictors of healthcare visits as missed opportunities for HIV diagnosis. AIDS. May 1, 2021;35(Suppl 1):S7-S18. [doi: ] [Medline: 33867485]

47.  Giron LB, Palmer CS, Liu Q, et al. Non-invasive plasma glycomic and metabolic biomarkers of post-treatment control of HIV. Nat Commun. Jun 29, 2021;12(1):3922. [doi: ] [Medline: 34188039]

48.  Murnane PM, Ayieko J, Vittinghoff E, et al. Machine learning algorithms using routinely collected data do not adequately predict viremia to inform targeted services in postpartum women living with HIV. J Acquir Immune Defic Syndr. Dec 15, 2021;88(5):439-447. [doi: ] [Medline: 34520443]

49.  Mahto R, Sood K. HIV progression and outcome prediction to enhance patient matching for clinical trials. Presented at: 2024 IEEE 14th Annual Computing and Communication Workshop and Conference (CCWC); Jan 8-10, 2024:0278-0284; Las Vegas, NV, USA. [doi: ]

50.  Prosperi M, Xu J, Guo JS, et al. Identification of social and racial disparities in risk of HIV infection in Florida using causal AI methods. Presented at: 2022 IEEE International Conference on Bioinformatics and Biomedicine (BIBM); Dec 6-8, 2022:2934-2939; Las Vegas, NV, USA. [doi: ]

51.  Lee JS, Paintsil E, Gopalakrishnan V, Ghebremichael M. A comparison of machine learning techniques for classification of HIV patients with antiretroviral therapy-induced mitochondrial toxicity from those without mitochondrial toxicity. BMC Med Res Methodol. Nov 27, 2019;19(1):216. [doi: ] [Medline: 31775643]

52.  Bose E, Paintsil E, Ghebremichael M. Minimum redundancy maximal relevance gene selection of apoptosis pathway genes in peripheral blood mononuclear cells of HIV-infected patients with antiretroviral therapy-associated mitochondrial toxicity. BMC Med Genomics. Dec 1, 2021;14(1):285. [doi: ] [Medline: 34852799]

53.  Premeaux TA, Bowler S, Friday CM, et al. Machine learning models based on fluid immunoproteins that predict non-AIDS adverse events in people with HIV. iScience. Jun 21, 2024;27(6):109945. [doi: ] [Medline: 38812553]

54.  Oliwa T, Furner B, Schmitt J, Schneider J, Ridgway JP. Development of a predictive model for retention in HIV care using natural language processing of clinical notes. J Am Med Inform Assoc. Jan 15, 2021;28(1):104-112. [doi: ] [Medline: 33150369]

55.  Ramachandran A, Kumar A, Koenig H, et al. Predictive analytics for retention in care in an urban HIV clinic. Sci Rep. Apr 14, 2020;10(1):6421. [doi: ] [Medline: 32286333]

56.  Petersen ML, LeDell E, Schwab J, et al. Super learner analysis of electronic adherence data improves viral prediction and may provide strategies for selective HIV RNA monitoring. JAIDS Journal of Acquired Immune Deficiency Syndromes. 2015;69(1):109-118. [doi: ]

57.  Lu Z, Ying H, Lin F, Neufeld S, Luborsky M, Brawn DM. Multi-class support vector machines for modeling HIV/AIDS treatment adherence using patient data. Presented at: International Joint Conference on Neural Networks 2005; Jul 31 to Aug 4, 2005:2417-2422; Montreal, Que, Canada. 2005.[doi: ]

58.  Ying H, Lin F, Luan X, et al. A fuzzy discrete event system with self-learning capability for HIV/AIDS treatment regimen selection. Presented at: NAFIPS 2005 - 2005 Annual Meeting of the North American Fuzzy Information Processing Society; Jun 26 to Feb 28, 2005:820-824; Detroit, MI, USA. 2005.[doi: ]

59.  Ying H, Lin F, MacArthur RD, et al. A fuzzy discrete event system approach to determining optimal HIV/AIDS treatment regimens. IEEE Trans Inf Technol Biomed. Oct 2006;10(4):663-676. [doi: ] [Medline: 17044400]

60.  Ying H, Lin F, MacArthur RD, et al. A self-learning fuzzy discrete event system for HIV/AIDS treatment regimen selection. IEEE Trans Syst Man Cybern B Cybern. Aug 2007;37(4):966-979. [doi: ] [Medline: 17702293]

61.  Goldbaum MH, Falkenstein I, Kozak I, et al. Analysis with support vector machine shows HIV-positive subjects without infectious retinitis have mfERG deficiencies compared to normal eyes. Trans Am Ophthalmol Soc. 2008;106:196-204. [Medline: 19277235]

62.  Cheng BY, Carbonell JG. Automatic detection of HIV drug resistance-associated mutations. Presented at: 2010 Ninth International Conference on Machine Learning and Application. Dec 12-14, 2010:IEEE. 528-533; NW Washington, DC, United States. 2010.[doi: ]

63.  Pillai SK, Pond SLK, Liu Y, et al. Genetic attributes of cerebrospinal fluid-derived HIV-1 env. Brain (Bacau). Jul 2006;129(7):1872-1883. [doi: ] [Medline: 16735456]

64.  Montesi G, Augello M, Polvere J, Marchetti G, Medaglini D, Ciabattini A. Predicting humoral responses to primary and booster SARS-CoV-2 mRNA vaccination in people living with HIV: a machine learning approach. J Transl Med. May 7, 2024;22(1):432. [doi: ] [Medline: 38715088]

65.  Underwood J, Cole JH, Caan M, et al. Gray and white matter abnormalities in treated human immunodeficiency virus disease and their relationship to cognitive function. Clin Infect Dis. Aug 1, 2017;65(3):422-432. [doi: ] [Medline: 28387814]

66.  Gelpi M, Mikaeloff F, Knudsen AD, et al. The central role of the glutamate metabolism in long-term antiretroviral treated HIV-infected individuals with metabolic syndrome. Aging (Milano). Oct 15, 2021;13(19):22732-22751. [doi: ]

67.  Olund Villumsen S, Benfeitas R, Knudsen AD, et al. Integrative lipidomics and metabolomics for system-level understanding of the metabolic syndrome in long-term treated HIV-infected individuals. Front Immunol. 2021;12:742736. [doi: ] [Medline: 35095835]

68.  Beerenwinkel N, Schmidt B, Walter H, et al. Diversity and complexity of HIV-1 drug resistance: a bioinformatics approach to predicting phenotype from genotype. Proc Natl Acad Sci U S A. Jun 11, 2002;99(12):8271-8276. [doi: ] [Medline: 12060770]

69.  Wang D, Larder B, Revell A, et al. A comparison of three computational modelling methods for the prediction of virological response to combination HIV therapy. Artif Intell Med. Sep 2009;47(1):63-74. [doi: ] [Medline: 19524413]

70.  Zazzi M, Incardona F, Rosen-Zvi M, et al. Predicting response to antiretroviral treatment by machine learning: the EuResist project. Intervirology. 2012;55(2):123-127. [doi: ] [Medline: 22286881]

71.  Heider D, Senge R, Cheng W, Hüllermeier E. Multilabel classification for exploiting cross-resistance information in HIV-1 drug resistance prediction. Bioinformatics. Aug 15, 2013;29(16):1946-1952. [doi: ] [Medline: 23793752]

72.  Riemenschneider M, Senge R, Neumann U, Hüllermeier E, Heider D. Exploiting HIV-1 protease and reverse transcriptase cross-resistance information for improved drug resistance prediction by means of multi-label classification. BioData Min. 2016;9:10. [doi: ] [Medline: 26933450]

73.  Riemenschneider M, Hummel T, Heider D. SHIVA - a web application for drug resistance and tropism testing in HIV. BMC Bioinformatics. Aug 22, 2016;17(1):314. [doi: ] [Medline: 27549230]

74.  Ramon E, Belanche-Muñoz L, Pérez-Enciso M. HIV drug resistance prediction with weighted categorical kernel functions. BMC Bioinformatics. Jul 30, 2019;20(1):410. [doi: ] [Medline: 31362714]

75.  Tunc H, Sari M, Kotil S. Machine learning aided multiscale modelling of the HIV-1 infection in the presence of NRTI therapy. PeerJ. 2023;11:e15033. [doi: ] [Medline: 37020854]

76.  Tunc H, Dogan B, Darendeli Kiraz BN, Sari M, Durdagi S, Kotil S. Prediction of HIV-1 protease resistance using genotypic, phenotypic, and molecular information with artificial neural networks. PeerJ. 2023;11:e14987. [doi: ] [Medline: 36967989]

77.  Paremskaia A, Rudik AV, Filimonov DA, Lagunin AA, Poroikov VV, Tarasova OA. Web service for HIV drug resistance prediction based on analysis of amino acid substitutions in main drug targets. Viruses. Nov 11, 2023;15(11):2245. [doi: ] [Medline: 38005921]

78.  Rojas Sánchez P, Cobos A, Navaro M, Ramos JT, Pagán I, Holguín Á. Impact of clinical parameters in the intrahost evolution of HIV-1 subtype B in pediatric patients: a machine learning approach. Genome Biol Evol. Oct 1, 2017;9(10):2715-2726. [doi: ] [Medline: 29044435]

79.  Ahlström MG, Ronit A, Omland LH, Vedel S, Obel N. Algorithmic prediction of HIV status using nation-wide electronic registry data. EClinicalMedicine. Dec 2019;17:100203. [doi: ] [Medline: 31891137]

80.  Bogojeska J, Bickel S, Altmann A, Lengauer T. Dealing with sparse data in predicting outcomes of HIV combination therapies. Bioinformatics. Sep 1, 2010;26(17):2085-2092. [doi: ] [Medline: 20624779]

81.  Zazzi M, Kaiser R, Sönnerborg A, et al. Prediction of response to antiretroviral therapy by human experts and by the EuResist data-driven expert system (the EVE study). HIV Med. Apr 2011;12(4):211-218. [doi: ] [Medline: 20731728]

82.  Rosen-Zvi M, Altmann A, Prosperi M, et al. Selecting anti-HIV therapies based on a variety of genomic and clinical factors. Bioinformatics. Jul 1, 2008;24(13):i399-406. [doi: ] [Medline: 18586740]

83.  Pham T, Ghafoor M, Grañana-Castillo S, et al. DeepARV: ensemble deep learning to predict drug-drug interaction of clinical relevance with antiretroviral therapy. NPJ Syst Biol Appl. May 6, 2024;10(1):48. [doi: ] [Medline: 38710671]

84.  Niemczak CE, Montagnese B, Levy J, et al. Machine learning for predicting cognitive deficits using auditory and demographic factors. PLoS ONE. 2024;19(5):e0302902. [doi: ] [Medline: 38743715]

85.  Singh Y. Machine learning to improve the effectiveness of ANRS in predicting HIV drug resistance. Healthc Inform Res. Oct 2017;23(4):271-276. [doi: ] [Medline: 29181236]

86.  Ekpenyong ME, Edoho ME, Udo IJ, et al. A transfer learning approach to drug resistance classification in mixed HIV dataset. Informatics in Medicine Unlocked. 2021;24:100568. [doi: ]

87.  Kimaina A, Dick J, DeLong A, Chrysanthopoulou SA, Kantor R, Hogan JW. Comparison of machine learning methods for predicting viral failure: a case study using electronic health record data. Stat Commun Infect Dis. Sep 1, 2020;12(Suppl1):20190017. [doi: ] [Medline: 37288469]

88.  Kagendi N, Mwau M. A machine learning approach to predict HIV viral load hotspots in Kenya using real-world data. Health Data Sci. 2023;3:0019. [doi: ] [Medline: 38487196]

89.  Mamo DN, Yilma TM, Tewelgne MF, et al. Machine learning to predict virological failure among HIV patients on antiretroviral therapy in the University of Gondar Comprehensive and Specialized Hospital, in Amhara Region, Ethiopia, 2022. BMC Med Inform Decis Mak. 2022;23(1):75. [doi: ]

90.  Bisaso KR, Karungi SA, Kiragga A, Mukonzo JK, Castelnuovo B. A comparative study of logistic regression based machine learning techniques for prediction of early virological suppression in antiretroviral initiating HIV patients. BMC Med Inform Decis Mak. Sep 4, 2018;18(1):77. [doi: ] [Medline: 30180893]

91.  Kebede M, Zegeye DT, Zeleke BM. Predicting CD4 count changes among patients on antiretroviral treatment: application of data mining techniques. Comput Methods Programs Biomed. Dec 2017;152:149-157. [doi: ] [Medline: 29054255]

92.  Ekpenyong ME, Etebong PI, Jackson TC. Fuzzy-multidimensional deep learning for efficient prediction of patient response to antiretroviral therapy. Heliyon. Jul 2019;5(7):e02080. [doi: ] [Medline: 31372545]

93.  Maskew M, Sharpey-Schafer K, De Voux L, et al. Applying machine learning and predictive modeling to retention and viral suppression in South African HIV treatment cohorts. Sci Rep. Jul 26, 2022;12(1):12715. [doi: ] [Medline: 35882962]

94.  Ogbechie MD, Fischer Walker C, Lee MT, et al. Predicting treatment interruption among people living with HIV in Nigeria: machine learning approach. JMIR AI. May 12, 2023;2:e44432. [doi: ] [Medline: 38875546]

95.  Tibúrcio R, Barreto-Duarte B, Naredren G, et al. Dynamics of T-lymphocyte activation related to paradoxical tuberculosis-associated immune reconstitution inflammatory syndrome in persons with advanced HIV. Front Immunol. 2021;12:757843. [doi: ] [Medline: 34691079]

96.  Ogishi M, Yotsuyanagi H. Prediction of HIV-associated neurocognitive disorder (HAND) from three genetic features of envelope gp120 glycoprotein. Retrovirology (Auckl). Jan 27, 2018;15(1):12. [doi: ] [Medline: 29374475]

97.  Babu H, Sperk M, Ambikan AT, et al. Plasma metabolic signature and abnormalities in HIV-infected individuals on long-term successful antiretroviral therapy. Metabolites. Sep 30, 2019;9(10):210. [doi: ] [Medline: 31574898]

98.  Yin Y, Xue M, Shi L, et al. A noninvasive prediction model for Hepatitis B virus disease in patients with HIV: based on the population of Jiangsu, China. Biomed Res Int. 2021;2021(1):6696041. [doi: ] [Medline: 33860053]

99.  Zhao J, Ma Z, Chen F, et al. Human immune deficiency virus-related structural alterations in the brain are dependent on age. Hum Brain Mapp. Jul 2021;42(10):3131-3140. [doi: ] [Medline: 33755269]

100.  Li B, Li M, Song Y, et al. Construction of machine learning models to predict changes in immune function using clinical monitoring indices in HIV/AIDS patients after 9.9-years of antiretroviral therapy in Yunnan, China. Front Cell Infect Microbiol. 2022;12:867737. [doi: ] [Medline: 35646738]

101.  Mulyadi WJ, Qomariyah NN. Using machine learning to analyse the effect of antiretroviral therapy (ART) on people with HIV. Presented at: 2023 10th International Conference on ICT for Smart Society (ICISS); Sep 6-7, 2023:1-5; Bandung, Indonesia. [doi: ]

102.  Jaganath D, Rajan J, Yoon C, et al. Evaluation of multi-antigen serological screening for active tuberculosis among people living with HIV. PLoS ONE. 2020;15(6):e0234130. [doi: ] [Medline: 32497095]

103.  Paul RH, Cho KS, Luckett P, et al. Machine learning analysis reveals novel neuroimaging and clinical signatures of frailty in HIV. J Acquir Immune Defic Syndr. Aug 1, 2020;84(4):414-421. [doi: ] [Medline: 32251142]

104.  Blassel L, Tostevin A, Villabona-Arenas CJ, et al. Using machine learning and big data to explore the drug resistance landscape in HIV. PLOS Comput Biol. Aug 2021;17(8):e1008873. [doi: ] [Medline: 34437532]

105.  Ragonnet-Cronin M, Golubchik T, Moyo S, et al. Human immunodeficiency virus (HIV) genetic diversity informs stage of HIV-1 infection among patients receiving antiretroviral therapy in Botswana. J Infect Dis. Apr 19, 2022;225(8):1330-1338. [doi: ] [Medline: 34077517]

106.  Streeck H, Maestri A, Habermann D, et al. Dissecting drivers of immune activation in chronic HIV-1 infection. EBioMedicine. Sep 2022;83:104182. [doi: ] [Medline: 35905559]

107.  Benitez AE, Musinguzi N, Bangsberg DR, et al. Super learner analysis of real-time electronically monitored adherence to antiretroviral therapy under constrained optimization and comparison to non-differentiated care approaches for persons living with HIV in rural Uganda. J Int AIDS Soc. Mar 2020;23(3):e25467. [doi: ] [Medline: 32202067]

108.  Stockman J, Friedman J, Sundberg J, Harris E, Bailey L. Predictive analytics using machine learning to identify ART clients at health system level at greatest risk of treatment interruption in Mozambique and Nigeria. JAIDS Journal of Acquired Immune Deficiency Syndromes. 2022;90(2):154-160. [doi: ]

109.  Herrera-Ibatá DM, Pazos A, Orbegozo-Medina RA, Romero-Durán FJ, González-Díaz H. Mapping chemical structure-activity information of HAART-drug cocktails over complex networks of AIDS epidemiology and socioeconomic data of U.S. counties. BioSystems. Jun 2015;132-133:20-34. [doi: ] [Medline: 25916548]
