# Supplementary material for: Machine Learning in HIV Care and Antiretroviral Therapy: Systematic Review
Source: J Med Internet Res. 2026 Apr 28;28:e79219. doi: 10.2196/79219 (PMC13123759; doi:10.2196/79219)
Supplement: Multimedia Appendix 3 [file jmir-v28-e79219-s003.docx]

**Top five algorithms and statistical methods for all categories**

| **Figure S1**  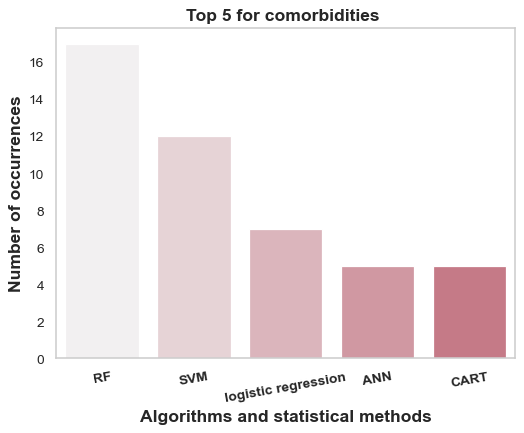 | **Figure S2**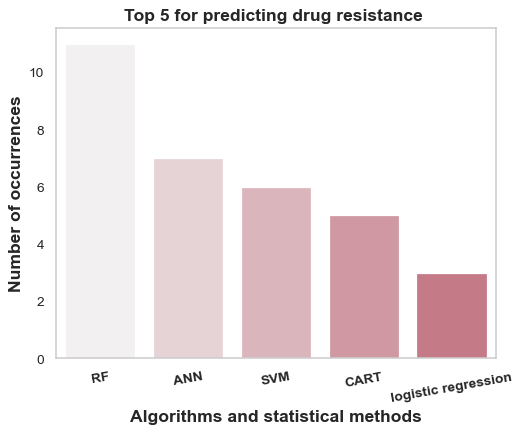 |
| --- | --- |
| **Figure S3**  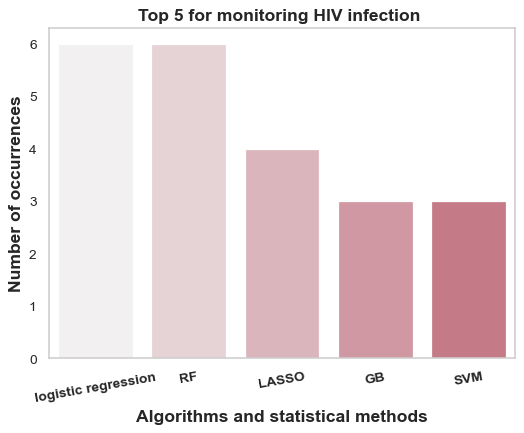 | **Figure S4**  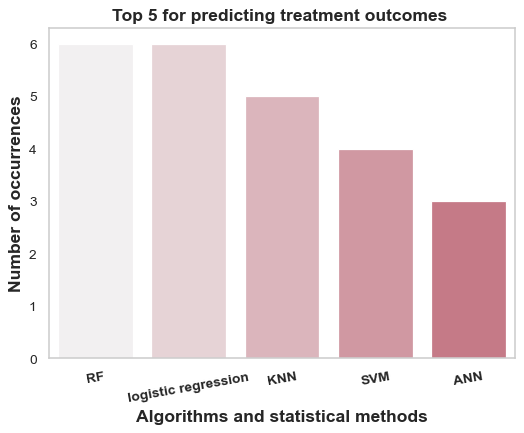 |
| **Figure S5**  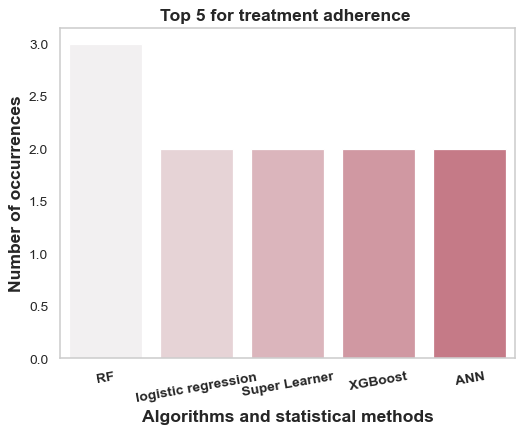 | **Figure S6**  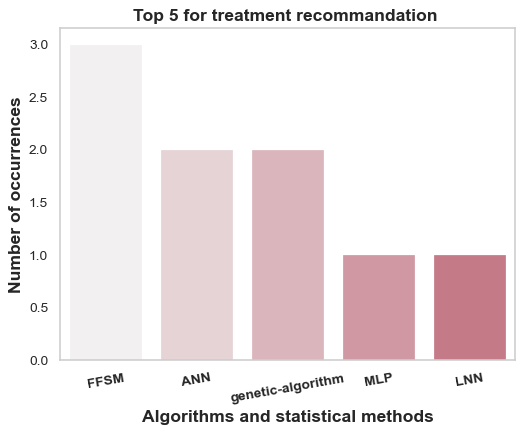 |
