# Supplementary material for: Machine Learning in HIV Care and Antiretroviral Therapy: Systematic Review
Source: J Med Internet Res. 2026 Apr 28;28:e79219. doi: 10.2196/79219 (PMC13123759; doi:10.2196/79219)
Supplement: Checklist 1 [file jmir-v28-e79219-s004.pdf]

PRISMA 2020 Checklist

Section and Topic	Item #	Checklist item	Location where item is reported
**TITLE**			
Title	1	Identify the report as a systematic review.<br>HIV and artificial intelligence: a systematic review of past applications, current trends and future perspectives.	Page 1
ABSTRACT			
Abstract	2	See the PRISMA 2020 for Abstracts checklist.<br>**Background:** Artificial Intelligence (AI) is expanding in various medical fields, with machine learning increasingly used to enhance patient management in diagnosis, prevention, and therapeutic care.<br>**Objective:** The aim of this study is to provide an overview of AI applications in HIV care, focusing on real clinical data to improve healthcare for people living with HIV and on antiretroviral therapy, while highlighting unexplored areas.<br>**Methods:** Following PRISMA 2020 guidelines, we analyzed four databases: PubMed, Embase, IEEE, and Web of Science. We excluded from this review studies (i) that were not directly focused on HIV or those that did not apply machine learning to real clinical data, (ii) that focused on pre-exposure prophylaxis (iii) study involving in silico antiretroviral drug development and (iv) studies on the biological mechanisms underlying HIV diagnosis.<br>**Results:** A total of 476 studies were identified, and after eligibility assessment, 98 were analyzed in detail. Random forests emerged as the most used algorithm, proving effective in identifying biomarkers of metabolic syndrome, genetic features of the HIV envelope, and predicting neurocognitive impairment. Support Vector Machines demonstrated strong abilities in analyzing the associations between HIV-1 genotypes and resistance phenotypes, predicting virological response to therapy based on HIV genotype, detecting mutations associated with HIV drug resistance without the need for expert knowledge, and enhancing computational predictions of resistance from genotype data. Logistic regression appears to be most powerful in predicting various treatment outcomes, including virological failure, adverse events, immune changes in patients receiving antiretrovirals, and biomarkers of mitochondrial toxicity.<br>**Conclusions:** Depending on the field of application, some Machine Learning methods are more suitable and adapt better to certain HIV concerns. However, some areas such as treatment recommendations, treatment adherence, and treatment optimization, still lack AI algorithms and need further exploration such as therapeutical optimization. The development of new clinical decision-support systems for people living with HIV is the new challenge for the years ahead, and AI represent one of the most promising tools to address it.	Page 1
**INTRODUCTION**			
Rationale	3	Describe the rationale for the review in the context of existing knowledge.<br>In recent years, Machine Learning has demonstrated its utility in optimizing and revolutionizing various aspects of healthcare and research. Two pivotal applications of AI for clinical purposes are Rule-Based Expert Systems and Clinical Decision Support Systems (CDSS). CDSS combines clinicians' medical expertise with recent AI advancements to enhance clinical decision-making processes. These systems leverage extensive medical knowledge derived from medical literature, complex algorithms, and patients' HER data to assist healthcare professionals in the overall quality of care while ensuring patient safety. This review aims to identify the main applications of ML in HIV/AIDS data management and to understand current trends.	Page 2
Objectives	4	Provide an explicit statement of the objective(s) or question(s) the review addresses.<br>The aim of this study is to provide an overview of AI applications in HIV care, focusing on real clinical data to improve healthcare for people living with HIV and on antiretroviral therapy, while highlighting unexplored areas. This review aims to identify the main applications of ML in HIV/AIDS data management and to understand current trends.	Page 3
**METHODS**			
Eligibility criteria	5	Specify the inclusion and exclusion criteria for the review and how studies were grouped for the syntheses.<br>Following PRISMA 2020 guidelines, we analyzed four databases: PubMed, Embase, IEEE, and Web of Science. We included studies which titles and abstracts mentioned explicitly searching terms (machine learning, antiretroviral therapy, and HIV) and using real clinical data. We excluded	Page 4

Section and Topic	Item #	Checklist item	Location where item is reported
		from this review studies:<br>• that were not directly focused on HIV or those that did not apply machine learning to real clinical data,<br>• that focused on pre-exposure prophylaxis, that centered on the pathophysiology of HIV infection,<br>• that involve *in silico* antiretroviral drug development, on the biological mechanisms underlying HIV diagnosis.<br>The included studies were grouped into six themes: comorbidities, prediction of drug resistance, monitoring of HIV infection, prediction of treatment outcome, treatment adherence and treatment recommendations.	
Information sources	6	Specify all databases, registers, websites, organisations, reference lists and other sources searched or consulted to identify studies. Specify the date when each source was last searched or consulted.<br>An extensive search was conducted across four major databases (PubMed, Embase, IEEE, and Web of Science), following the PRISMA 2020 guidelines, without applying any date restrictions, with the combination of keywords based on a controlled vocabulary thesaurus for indexing articles in each database: “Machine Learning” and “HIV” and “Antiretroviral Therapy”. The databases were last consulted on 31 August 31 2024.	Page 4
Search strategy	7	Present the full search strategies for all databases, registers and websites, including any filters and limits used.<br>We selected studies using the following search terms: machine learning, antiretroviral therapy, and HIV on four databases. Four hundred and seventy-six studies were collected: 148 from PubMed, 200 from Embase, 16 from IEEE, and 112 from Web of Science.	Page 4
Selection process	8	Specify the methods used to decide whether a study met the inclusion criteria of the review, including how many reviewers screened each record and each report retrieved, whether they worked independently, and if applicable, details of automation tools used in the process.<br>Four hundred and seventy-six studies were collected: 148 from PubMed, 200 from Embase, 16 from IEEE, and 112 from Web of Science. Studies were included in the current review if they met all the following criteria: titles and abstracts which mentioned explicitly searching terms and using real clinical data. Non-inclusion criterions were studies on *in silico* drug design of ART, studies on pre-exposure prophylaxis, research focused on the pathophysiology of HIV infection, and papers discussing advancements in HIV clinical diagnostics. During the initial screening, 331 records were excluded due to not meeting the research criteria, such as a lack of relevance to HIV or the absence of machine learning applied to clinical data. Out of 145 articles that advanced to the eligibility phase, 16 were excluded due to retrieval issues, including articles that were literature reviews or meta-analyses. After successfully retrieving 129 articles, 31 duplicates were eliminated, resulting in 98 articles that fully met the guidelines and inclusion criteria for this review. The retrieved articles were grouped into six key themes: comorbidities, predicting drug resistance, monitoring HIV infection, predicting treatment outcomes, treatment adherence and treatment recommendations. Three reviewers screened each articles independently, grouping them into these six key themes. In the event of disagreement, a joint proofreading was carried out to reach a consensus.	Page 5
Data collection process	9	Specify the methods used to collect data from reports, including how many reviewers collected data from each report, whether they worked independently, any processes for obtaining or confirming data from study investigators, and if applicable, details of automation tools used in the process.<br>We compiled a database that includes the essential details of each study: study title, year of publication, authors, geographical location and key thematic category, such as comorbidities, prediction of resistance or treatment adherence, with sub-categories for further classification. In addition, we have extracted data on the types of information analyzed (e.g. clinical, biological) and the data format (numerical, categorical, textual). The database also documents study objectives, main results, employed AI algorithms and methods, sample size and authors countries of origin. For data trends analysis, concerning the origin of the articles, the origin of the first author was chosen.	Page 5
Data items	10a	List and define all outcomes for which data were sought. Specify whether all results that were compatible with each outcome domain in each study were sought (e.g. for all measures, time points, analyses), and if not, the methods used to decide which results to collect.<br>All results were sought and collected	Page 6

PRISMA 2020 Checklist

Section and Topic	Item #	Checklist item	Location where item is reported
	10b	List and define all other variables for which data were sought (e.g. participant and intervention characteristics, funding sources). Describe any assumptions made about any missing or unclear information.<br>Variables: year: Year of publication.<br>Author: Author's name.<br>Geographic localization: Location of the affiliated research laboratory.<br>Title: Article's complete title.<br>Category: The assigned category (Comorbidities, Monitoring HIV infection, Predicting drug resistance, Predicting treatment outcomes, Treatment adherence, Treatment outcomes).<br>Subcategory: The subcategory assigned withing the main category.<br>AI: The artificial intelligence employed.<br>Statistical analysis: Statistical methods employed.<br>Data: Data type.<br>Number of references: Number of articles referenced in each article.<br>Purpose: Objective of the article.<br>Findings: The results discussed in the article.	Page [6-13]
Study risk of bias assessment	11	Specify the methods used to assess risk of bias in the included studies, including details of the tool(s) used, how many reviewers assessed each study and whether they worked independently, and if applicable, details of automation tools used in the process.<br>All reviews assessed each study.	Page [6-13]
Effect measures	12	Specify for each outcome the effect measure(s) (e.g. risk ratio, mean difference) used in the synthesis or presentation of results.<br>Not applicable	/
Synthesis methods	13a	Describe the processes used to decide which studies were eligible for each synthesis (e.g. tabulating the study intervention characteristics and comparing against the planned groups for each synthesis (item #5)).<br>To determine which studies were eligible for each synthesis, we first extracted and tabulated the main characteristics of all included studies, such as title, abstract, results, and systematically compared them with the six predefined groups described in item#5.	
	13b	Describe any methods required to prepare the data for presentation or synthesis, such as handling of missing summary statistics, or data conversions.<br>The extracted data were cleaned and prepared for synthesis using several Python libraries, including numpy, pandas, re, scipy.stats, math, seaborn, matplotlib.pyplot, and wordcloud. Exploratory analyses and visualizations were performed to identify potential anomalies or outliers, and textual data were normalized using regular expressions. The entire process was documented for reproducibility.	
	13c	Describe any methods used to tabulate or visually display results of individual studies and syntheses.<br>To visually display the results of individual studies and syntheses, we used a combination of structured tables and graphical representations created with Python libraries such as pandas, seaborn, and matplotlib.pyplot.	
	13d	Describe any methods used to synthesize results and provide a rationale for the choice(s). If meta-analysis was performed, describe the model(s), method(s) to identify the presence and extent of statistical heterogeneity, and software package(s) used.<br>Results were synthesized using narrative and descriptive methods, as no meta-analysis was conducted. The synthesis focused on comparing outcomes across studies based on methodological approaches, performance metrics, and study contexts.	

PRISMA 2020 Checklist

Section and Topic	Item #	Checklist item	Location where item is reported
	13e	Describe any methods used to explore possible causes of heterogeneity among study results (e.g. subgroup analysis, meta-regression).<br>None	
	13f	Describe any sensitivity analyses conducted to assess robustness of the synthesized results.<br>None	
Reporting bias assessment	14	Describe any methods used to assess risk of bias due to missing results in a synthesis (arising from reporting biases).<br>None	
Certainty assessment	15	Describe any methods used to assess certainty (or confidence) in the body of evidence for an outcome.<br>None	
**RESULTS**			
Study selection	16a	Describe the results of the search and selection process, from the number of records identified in the search to the number of studies included in the review, ideally using a flow diagram.	Page [6-13]

<table>
<tr><th>Section and Topic</th><th>Item #</th><th>Checklist item</th><th>Location where item is reported</th></tr>
<tr><td></td><td></td><td>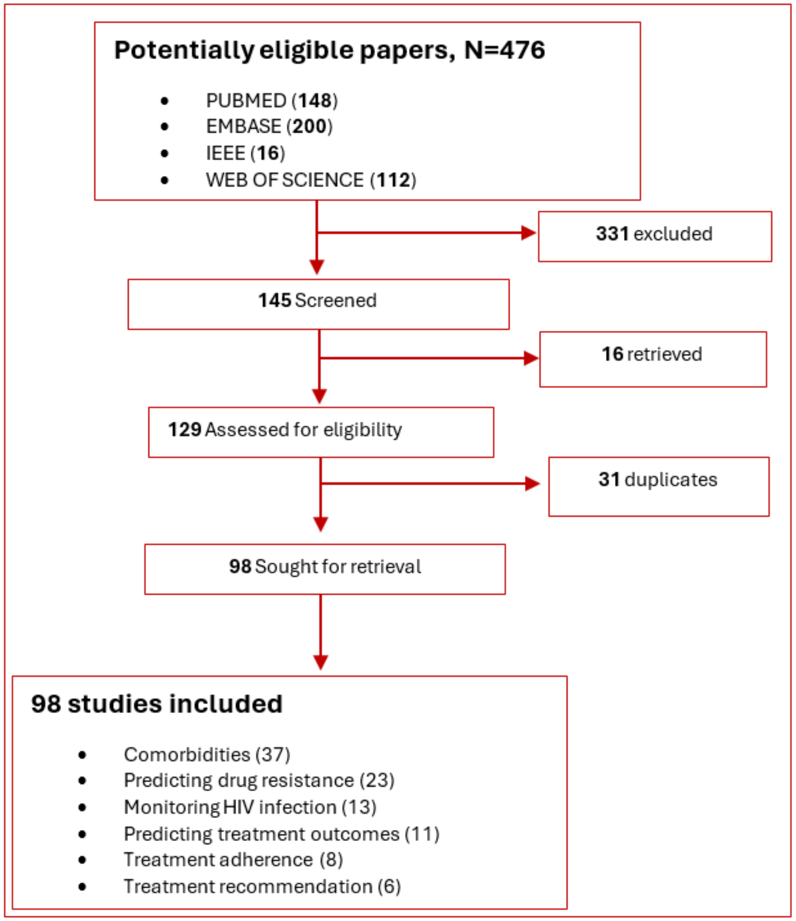
</td><td></td></tr>
<tr><td></td><td>16b</td><td>Cite studies that might appear to meet the inclusion criteria, but which were excluded, and explain why they were excluded.
Non-inclusion criterions were studies on in silico drug design of ART, studies on pre-exposure prophylaxis, research focused on the pathophysiology of HIV infection, and papers discussing advancements in HIV clinical diagnostics. During the initial screening, 331 records were excluded due to not meeting the research criteria, such as a lack of relevance to HIV or the absence of machine learning applied to clinical data. Out of 145 articles that advanced to the eligibility phase, 16 were excluded due to retrieval issues, including articles that were literature reviews or meta-analyses. After successfully retrieving 129 articles, 31 duplicates were eliminated, resulting in 98 articles that fully met the guidelines and inclusion criteria for this review. The retrieved articles were grouped into six key themes: comorbidities, predicting drug resistance, monitoring HIV infection, predicting treatment outcomes, treatment adherence and treatment recommendations.</td><td></td></tr>
<tr><td>Study</td><td>17</td><td>Cite each included study and present its characteristics.</td><td></td></tr>
</table>

PRISMA 2020 Checklist

Section and Topic	Item #	Checklist item	Location where item is reported
characteristics		A detailed list of all included studies, along with their key characteristic such as authors, year of publication, title, purpose and findings are provided in Appendix 2.	
Risk of bias in studies	18	Present assessments of risk of bias for each included study.<br>Reviewers evaluated all studies using a structured assessment strategy, and disagreements were resolved by consensus.	
Results of individual studies	19	For all outcomes, present, for each study: (a) summary statistics for each group (where appropriate) and (b) an effect estimate and its precision (e.g. confidence/credible interval), ideally using structured tables or plots.<br>The outcomes of each study are summarized in Appendix 2.	
Results of syntheses	20a	For each synthesis, briefly summarise the characteristics and risk of bias among contributing studies.<br>In summary, our review identified the most suitable Machine Learning methods across the six categories chosen. Random Forest emerged as the most used algorithm due to its versatility and suitability for both classification and prediction tasks. Following closely was the Support Vector Machine, both of which are supervised machine learning algorithms widely applied to various aspects of HIV infection challenges.	
	20b	Present results of all statistical syntheses conducted. If meta-analysis was done, present for each the summary estimate and its precision (e.g. confidence/credible interval) and measures of statistical heterogeneity. If comparing groups, describe the direction of the effect.<br>We started our analysis with the literature trends by doing a statistical analysis of publication years which revealed that, on average, articles were published in 2018, with a standard deviation of 5.63 years. The quartiles provide additional insight: the first quartile indicates that 25% of articles were published before 2015. The median year of publication is 2020, meaning that half the articles were published after this date. Finally, 25% of articles were published after 2020, highlighting a clear trend in favor of more recent studies. These results reflect a strong temporal coverage of our literature review. Concerning the paper distribution over time, the earliest study on HIV treatment using AI appeared in 2002, and while publications remained sporadic until 2005, there was a noticeable uptick afterwards.	
	20c	Present results of all investigations of possible causes of heterogeneity among study results.<br>None	
	20d	Present results of all sensitivity analyses conducted to assess the robustness of the synthesized results.<br>None	
Reporting biases	21	Present assessments of risk of bias due to missing results (arising from reporting biases) for each synthesis assessed.<br>None	
Certainty of evidence	22	Present assessments of certainty (or confidence) in the body of evidence for each outcome assessed.<br>None	
**DISCUSSION**			
Discussion	23a	Provide a general interpretation of the results in the context of other evidence.<br>Some AI methods are more suitable and adapt better to certain HIV concerns.	Pages [13-17]
	23b	Discuss any limitations of the evidence included in the review.<br>The diversity of databases is a critical element to consider. However, key challenges remain, including limited data availability, quality, and accessibility, which continue to hinder the broader adoption of evidence-based medicine in HIV care. Although there has been an increase in publications across various aspects of HIV care, there remains a critical lack of research addressing treatment safety and optimization.	
	23c	Discuss any limitations of the review processes used.<br>Several limitations may have influenced the comprehensiveness and consistency of this review. First, despite efforts to conduct a thorough search, it is possible that some relevant studies were missed due to publication bias, incomplete indexing, or the exclusion of non-English	Page [13-17]

PRISMA 2020 Checklist

Section and Topic	Item #	Checklist item	Location where item is reported
		literature. Second, the assessment of risk of bias and study eligibility relied on subjective judgment, even though multiple reviewers were involved, and disagreements were resolved by consensus.	
	23d	Discuss implications of the results for practice, policy, and future research.<br>Future efforts should focus on monitoring adverse events and developing decision-support tools based on the risk of drug-related adverse effects. Addressing this gap will be the focus for future studies, where the aim to leverage Artificial Intelligence is to develop more personalized, effective ART regimens.	Page [13-17]
**OTHER INFORMATION**			
Registration and protocol	24a	Provide registration information for the review, including register name and registration number, or state that the review was not registered.<br>The review was not registered	
	24b	Indicate where the review protocol can be accessed, or state that a protocol was not prepared.<br>A protocol was not prepared.	
	24c	Describe and explain any amendments to information provided at registration or in the protocol.<br>No amendments.	
Support	25	Describe sources of financial or non-financial support for the review, and the role of the funders or sponsors in the review.<br>This work was funded by the Burgundy Franche-Comté region.	
Competing interests	26	Declare any competing interests of review authors.<br>Jennifer Lagoutte-Renosi received fees to attend meetings from Viiv healthcare, Janssen-Cilag, Gilead Sciences and Pfizer. The other authors declare no competing interests.	
Availability of data, code and other materials	27	Report which of the following are publicly available and where they can be found; template data collection forms; data extracted from included studies; data used for all analyses; analytic code; any other materials used in the review.<br>Data used in the analysis are available in the appendix 2. Please contact Dr. Jennifer LAGOUTTE RENOSI via email for data inquiries.	

From: Page MJ, McKenzie JE, Bossuyt PM, Boutron I, Hoffmann TC, Mulrow CD, et al. The PRISMA 2020 statement: an updated guideline for reporting systematic reviews. BMJ 2021;372:n71. doi: 10.1136/bmj.n71
